# A Platform for Screening Potential Anticholinesterase Fractions and Components Obtained from *Anemarrhena asphodeloides* Bge for Treating Alzheimer's Disease

**DOI:** 10.1155/2014/524650

**Published:** 2014-04-17

**Authors:** Yu Sun, Ying Peng, Lin-Guang Li, Li-Wei Zheng, Dong-Ju Lin, Ling-Zhi Li, Shao-Jiang Song

**Affiliations:** ^1^School of Traditional Chinese Materia Medica, Shenyang Pharmaceutical University, Shenyang, Liaoning 110016, China; ^2^School of Pharmacy, Shenyang Pharmaceutical University, Shenyang, Liaoning 110016, China; ^3^Key Laboratory of Structure-Based, Drug Design & Discovery, Ministry of Education, Shenyang, Liaoning 110016, China

## Abstract

Alzheimer's disease (AD) is a neurodegenerative disease characterized by progressive memory loss and cognitive impairment. Cholinesterase inhibitors are widely used for the symptomatic treatment of Alzheimer's disease to enhance central cholinergic transmission. In this study, a bioactivity-oriented screening platform based on a modified Ellman's method and HPLC-QTOF MS technique was developed to rapidly screen active agents of *Anemarrhena asphodeloides* Bge. The 60% ethanol fraction from an ethyl acetate extract exhibited the most potential anticholinesterase activity. Fifteen steroid saponins were identified by the mass spectrum, standards and literature reports. Twenty-five compounds were isolated from the active fraction. The results showed that compounds with the C_6_–C_3_–C_6_ skeleton probably had both AChE and BuChE inhibitory activities. Xanthone and benzene derivatives exhibited no or little activity. Lignans showed weak BuChE inhibitory activity. The steroidal saponins demonstrated moderate or weak AChE inhibitory activity.

## 1. Introduction

Alzheimer's disease (AD) is a progressive neurodegenerative disorder of the central nervous system (CNS), characterized by deposits of aberrant proteins, namely, *β*-amyloid (A*β*) and *τ*-protein, loss of synapses, death of cholinergic neurons, and oxidative stress [1, 2]. The etiopathogenesis of AD still remains unknown, although the neurodegeneration leads to remarkable reduction of neurotransmitter acetylcholine at the synaptic clefts [3, 4]. An effective strategy to slow down the progression of deterioration in AD patients is using anticholinesterase inhibitors.

Acetylcholinesterase (AChE) and butyrylcholinesterase (BuChE) constitute the group of cholinesterases. AChE hydrolyses acetylcholine (ACh) and is mainly associated with nerves and muscles, being typically found on the synapses. AChE inhibitors are widely used for the symptomatic treatment of AD to enhance central cholinergic transmission. BuChE hydrolyses butyrylcholine (BuCh) and is synthesized by the liver, being found in large concentration in serum [5]. In healthy brains, AChE hydrolyzes the majority of ACh while BuChE plays a secondary role. However, as AD progresses, BuChE can compensate for AChE when the activity of AChE is inhibited by AChE inhibitors. Thus, BuChE hydrolyses the already depleted levels of ACh in these patients [6]. It has been proposed that individuals with low activity of BuChE can sustain cognitive functions better than individuals with normal BuChE activity. Furthermore, BuChE inhibitors have been reported to produce a significant increase in brain extracellular AChE without triggering severe peripheral or central side effects [7].

*Anemarrhena asphodeloides* Bge, which belongs to the family Liliaceae, is widely distributed in China [8]. The rhizomes of* Anemarrhena asphodeloides* Bge have been reported to have the cholinesterase inhibitory activity relevant to treatment of AD [9]. In order to find the candidate of a drug treating AD, the activities of fractions and compounds were assayed. Screening and identification of bioactive constituents in traditional Chinese medicines (TCMs) are the keys of the development of TCMs [10, 11]. However, the complexity and variability of TCMs present a challenge to the identification of their structures. Now more and more attention has been attracted to bioactivity-based LC-MS/MS identification technology owing to its high efficiency and high specificity [12, 13].

In this work, we used a bioactivity-oriented screening strategy, which was based on a modified Ellman's method and high performance liquid chromatography quadrupole-time-of-flight mass spectrometer (HPLC-QTOF MS) technique. The 60% ethanol fraction from an ethyl acetate extract showed the most potential anticholinesterase activity. Fifteen steroid saponins were identified by the mass spectrum, standards, and literature reports. Twenty-five compounds were isolated from the active fraction. Compounds with the C_6_–C_3_–C_6_ skeleton probably had both AChE and BuChE inhibitory activities. Xanthone and benzene derivatives exhibited no or little activity. Lignans showed weak BuChE inhibitory activity. The steroidal saponins demonstrated moderate or weak AChE inhibitory activity.

## 2. Materials and Methods

### 2.1. Plant Material

The rhizomes of* Anemarrhena asphodeloides* Bge were purchased from Beijing Tongrentang pharmacy. The plant materials were identified by Professor Jincai Lu, Department of Traditional Chinese Materia Medica, Shenyang Pharmaceutical University.

### 2.2. General Instrumental Equipment

HPLC system (Agilent, USA) consisted of a model G1276A pump, model G1367B Autosampler and model G1316A UV detector. The chromatograph was equipped with a reversed-phase C18 column of Grace Alltima (250 mm × 4.6 mm, 5 *μ*m). The QTOF-MS system (Bruker, Germany) with an ESI source was performed. HPLC separations were performed on a Hitachi 655-15 series pumping system equipped with a Hitachi L-2490 refractive index detector using a YMC-Park ODS-A column (250 × 10 mm I.D, S-5 *μ*m, and 12 nm). NMR spectra were performed on a Bruker ARX-300, ARX-400, and ARX-600 spectrometer using trimethylchlorosilane as the internal standard. Column chromatography was performed on a 200–300 mesh silica gel (Qingdao Marine Chemical Factory, People's Republic of China). Column chromatography was performed using YMC ODS-A gel (12 nm S-75 *μ*m, YMC Co., Ltd., Japan) and D-101 macroporous adsorption resin (Shanghai Hualing Resin Factory, People's Republic of China). TLC was performed with precoated silica gel GF_254_ plates (Qingdao Marine Chemical Factory, People's Republic of China). Microplate reader (Thermofisher Scientific, Finland) was used to test the activities. Acetylthiocholine iodide, S-butyrylthiocholine iodide, AChE, and BuChE were bought from Sigma Company.

### 2.3. Extraction Procedures

Dried rhizomes of* Anemarrhena asphodeloides* Bge were powdered into a homogeneous size by a disintegrator and then sieved (60 mesh). The materials were extracted by three different techniques (ultrasonic, heat reflux, and cold soak techniques). Various solvents including petroleum ether, dichloromethane, ethyl acetate, acetone, methanol, 95% ethanol, 70% ethanol, 50% ethanol, 30% ethanol, and water were used for preparing active fractions. Accurate 5.0 g of the* Anemarrhena asphodeloides* Bge powders was weighted into ten Erlenmeyer flasks containing the mentioned ten solvents of 500 mL individually and then extracted with ultrasonic-assisted method twice (30 min each). After filtering, the filtrates were amalgamated and evaporated to dryness by rotary evaporator (50°C). For the heat reflux, the powders of 5.0 g were soaked in different solvents of 50 mL each for 30 min. Then, the heated reflux extraction experiments were conducted in water bath (90°C) for 2 h. The solutions were filtrated when they were still hot. Extractions were carried out for three times and the filtrates were evaporated to dryness. Lastly, 5.0 g of the powders was accurately weighed and soaked in 500 mL solvents overnight and then evaporated the filtrates to dryness.

### 2.4. AChE and BuChE Inhibitory Assay

Cholinesterase inhibitory activity was evaluated using the modified method of Ellman. For AChE inhibitory assay, the reaction mixture consisted of 50 *μ*L of 100 mM PBS, pH 8.0, 25 *μ*L of 15 mM acetylthiocholine iodide, and 25 *μ*L of agents (1 mg/mL for extracts, 0.1 mg/mL for compounds) in a 96-well plate. The mixture was preincubated in 4°C for 10 min, and after that, 25 *μ*L of AchE (0.226 U/mL) and 125 *μ*L of 3 mM DTNB were added. The reactions were kept in 37°C for 20 min and then scanned at 412 nm with a microplate reader. For BuChE inhibitory assay, the same procedures were followed except for the use of substrate and enzyme, and S-butyrylthiocholine iodide and BuchE were used, respectively [14]. All of the experiments were repeated at least three times.

### 2.5. Structure Characterization and Identification of Active Fraction

The ethyl acetate extract dealt with by the ultrasonic method was applied to a D-101macroporous resin column and eluted with ethanol and water to give 0%, 20%, 40%, 60%, 80%, and 95% ethanol fractions. The constituents of active fraction were assayed by HPLC-QTOF-MS. HPLC system (Agilent, USA) consisted of a model G1276A pump, a model G1367B Autosampler, and a model G1316A UV detector. The chromatograph was equipped with a reversed-phase C18 column of Grace Alltima (250 mm × 4.6 mm, 5 *μ*m) eluted with a gradient mobile phase. Mobile phases were water with 0.1% of formic acid (A) and acetonitrile (B). The gradient used was as follows: 0 min, 5% B; 0–10 min, 5% to 15% B; 10–18 min, 15% to 20% B; 18–23 min, 20% to 23% B; 23–30 min, 23% to 25% B; 30–48 min, 25% to 30% B; 48–55 min, 30% to 50% B; 55–80 min, 50% to 100% B. The injection volume of sample was 5 *μ*L. The flow rate was 0.8 mL min^−1^ and the column temperature was ambient temperature. The QTOF-MS system (Bruker, Germany) with an ESI source was performed in positive mode. The parameters of ESI-MS were set as follows: the capillary voltage (+3800 V), the nebulizer gas pressure (1.2 bar), the dry gas flow rate (8.0 L min^−1^), and temperature (180°C). MS conditions were corrected by direct infusion of sodium formic acid solution (1 mM L^−1^) delivered by a syringe pump at a flow rate of 3 *μ*L min^−1^. The data were analyzed by Bruker Daltonics Data Analysis 3.4 software.

### 2.6. Isolation and Purification of Compounds from* Anemarrhena asphodeloides* Bge

**Figure 1:**
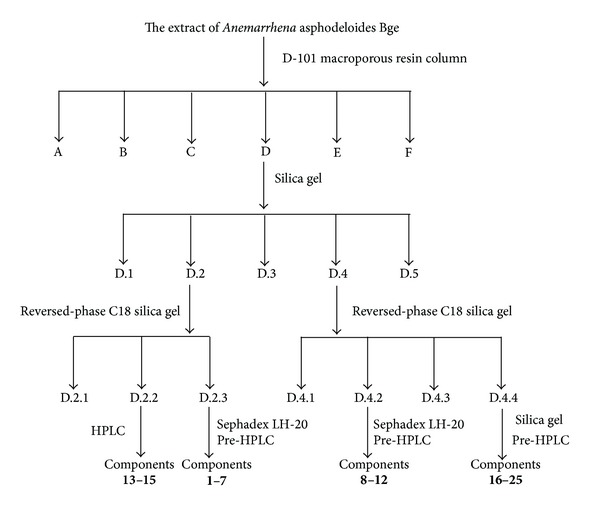
The isolation procedure of* Anemarrhena asphodeloides* Bge.

The extract of* Anemarrhena asphodeloides* Bge (400.0 g) was subjected to CC (macroporous adsorption resin D-101; gradient EtOH/H_2_O 0 : 100 to 95 : 5) to afford six fractions (*Frs*.* A*–*F*).* Fr*.* D* (62.0 g) was subjected to CC (silica gel; CH_2_Cl_2_/MeOH 100 : 0 to 0 : 100) and afforded five fractions (*Frs. D.1*–*D.5*).* Fr. D.2* (5.2 g) was subjected to CC (reversed-phase C_18_ silica gel; MeOH/H_2_O 30 : 70 to 100 : 0) to afford three fractions (*Frs*.* D.2.1*–*D.2.3*).* Fr. D.2.2* (0.7 g) was further purified by RP-HPLC with MeOH/H_2_O as mobile phase (85 : 15) to afford compounds** 13** to** 15**.* Fr. D.3* (2.3 g) was subjected to CC (Sephadex* LH-20*; MeOH) and then purified by RP-HPLC with MeOH/H_2_O as mobile phase (80 : 10) to afford compounds** 1** to** 7**.* Fr. D.4* (21.0 g) was subjected to CC (reversed-phase C_18_ silica gel; MeOH/H_2_O 30 : 70 to 100 : 0) and afforded four fractions.* Fr. D.4.2* (3.3 g) was subjected to CC (Sephadex* LH-20*; MeOH) and then purified by RP-HPLC with MeOH/H_2_O as mobile phase (50 : 50) to afford compounds** 8** to** 12**.* Fr. D.4.4* (9.7 g) was subjected to CC (silica gel; CH_2_Cl_2_/MeOH 30 : 70 to 100 : 0) and then purified by RP-HPLC with MeOH/H_2_O as mobile phase (70 : 30) to afford compounds** 16** to** 25**. The isolation procedure of* Anemarrhena asphodeloides* Bge is shown in Figure 1.

### 2.7. Statistical Analysis

Data were expressed as means ± S8EM and analyzed statistically by one-way ANOVA, followed by post hoc (LSD) test. The results were considered statistically significant at *P* value <0.05.

## 3. Results

### 3.1. The Activities of the Extracts

Screening the extracts of* Anemarrhena asphodeloides* Bge dealing with three different techniques and ten kinds of solvents showed that all the extracts demonstrated no AchE inhibitory activity but exhibited BuChE inhibitory activity. The extract dealing with dichloromethane, ethyl acetate, and acetone had much more inhibition ratio than the others. The ultrasonic method and the cold soak method were better than the heat reflux method. It was found that* Anemarrhena asphodeloides* Bge extracted with ethyl acetate by the ultrasonic technique had the most potential anticholinesterase activity. The results on the effects of the tested extracts on AChE and BuChE inhibitory activities were summarized in Table 1.

### 3.2. Screening the Active Fraction

**Figure 2:**
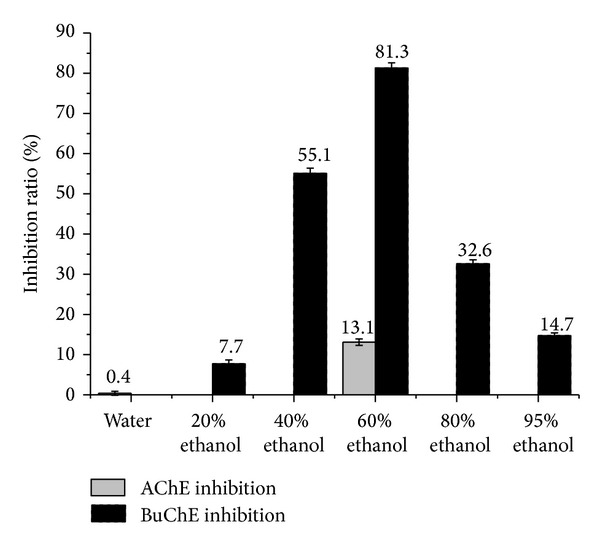
The effects of ethanol and water fractions on AChE and BuChE inhibitory activities.

The extract dealing with ethyl acetate by the ultrasonic method was applied to a D-101 macroporous resin column and eluted with ethanol and water to give 0%, 20%, 40%, 60%, 80%, and 95% ethanol fractions. Then they were tested on AChE and BuChE inhibitory activities. It indicated that the extract was partitioned into six fractions and active compounds were concentrated into 60% ethanol fraction. The results were summarized in Figure 2. Donepezil was used as positive drug with the inhibition ratio of 98.2% and 79.8%.

### 3.3. Structure Characterization and Identification of the Active Fraction

**Figure 3:**
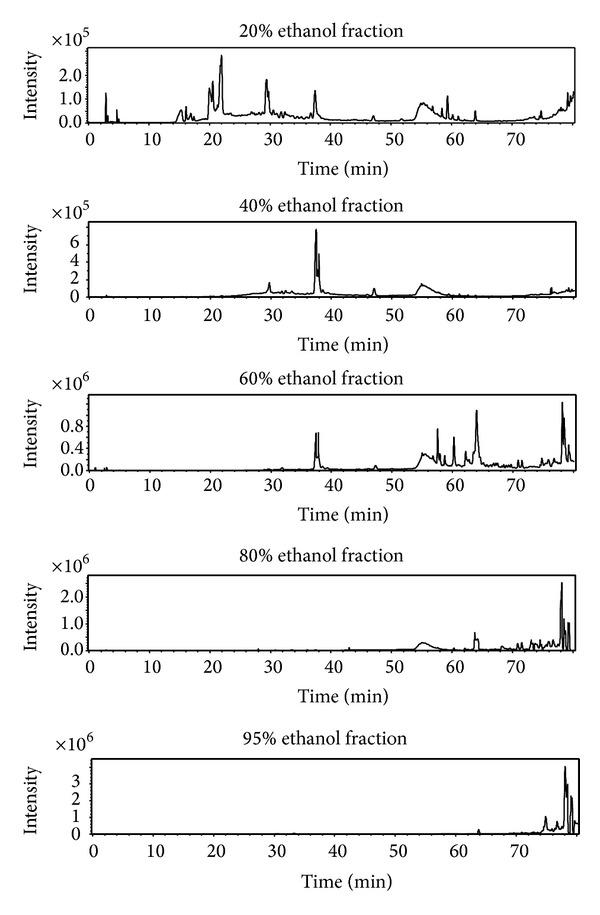
The total ion chromatograms of the five fractions.

The 20%, 40%, 60%, 80%, and 95% ethanol fractions were analyzed by HPLC-QTOF-MS technique. The water fraction was not tested for its inactivity. The total ion chromatograms of the five fractions were shown in Figure 3. As summarized in Table 2, a total of fifteen saponins from the 60% ethanol fraction were identified and tentatively characterized.

**Figure 4:**
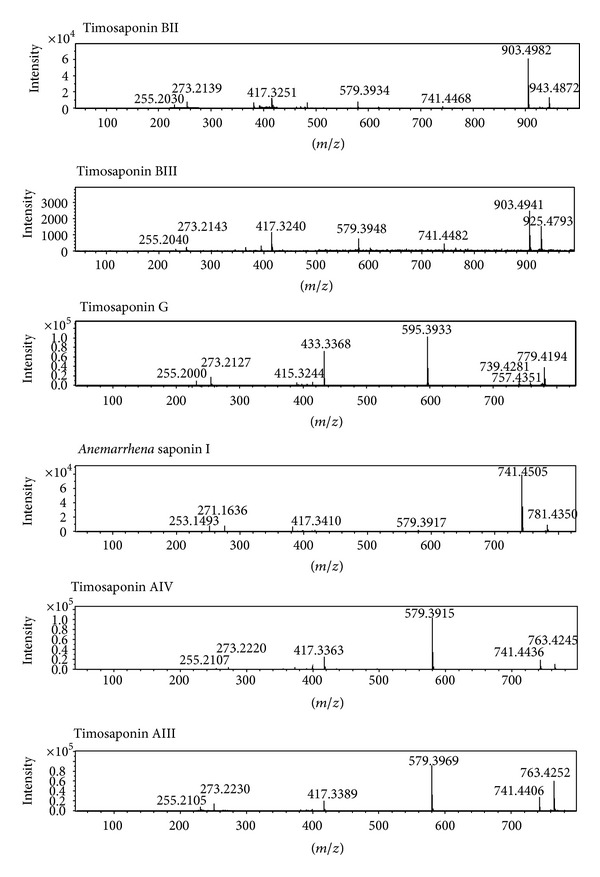
MS spectra of the authentic standards.

To obtain the information about precursor ions and characteristic fragment ions of the compounds, six authentic standards were injected into the LC-MS system. The fragmentation patterns for these authentic standards were discussed in detail below. MS spectra of the authentic standards were shown in Figure 4.

**Figure 5:**
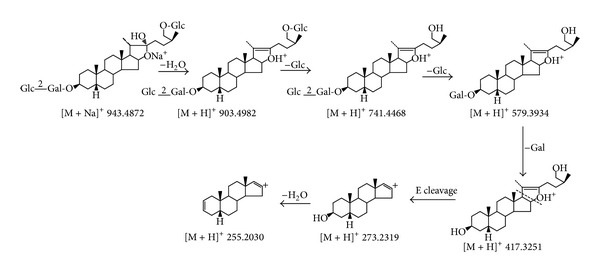
The proposed ESI-MS/MS fragmentation pathway of timosaponin BII.

Timosaponin BII produced a precursor ion [M + Na]^+^ at *m*/*z* 943.4872 (C_45_H_76_O_19_Na). As the C22 position was an active site, it readily eliminated the 22-hydroxyl residue to produce the thermal degradation ion [M + H – H_2_O]^+^ at *m*/*z* 903.4982 (C_45_H_75_O_18_), which could generate an ion at *m*/*z* 741.4468 [M + H – H_2_O – 162]^+^ by further neutral loss of a glucose moiety from the C26 position. At the same time, the product ions at 579.3934 [M + H – H_2_O – 2×162]^+^ and 417.3251 [M + H – H_2_O – 3 × 162]^+^, which corresponded to skeleton residues by successive losses of terminal hexose, could also be clearly observed. Furthermore, the fragment ion at *m*/*z* 273.2139 [417.3251 – C_8_H_16_O_2_]^+^ was attributed to a skeleton residue by the cleavage of the C20–C22 and C17–C20 bonds due to the presence of a 16,22-epoxy residue and a 22,26-epoxy residue, and then the ion continued to lose H_2_O to yield an ion at *m*/*z* 255.2030 [273.2139 – H_2_O]^+^. Major fragmentations proposed for timosaponin BII were presented in Figure 5.

Timosaponin BIII gave an [M + H]^+^ ion at *m*/*z* 903.4941 and an [M + Na]^+^ ion at *m*/*z* 925.4793, corresponding to an elemental composition C_45_H_74_O_18_. Its successive losses of one galactosyl and two glucosyls from the protonated ion led to the product ions at *m*/*z* 741.4482 [M + H – 162]^+^, 579.3948 [M + H – 2 × 162]^+^, and 417.3240 [M + H – 3 × 162]^+^. In addition, the fragment ions found at *m*/*z* 273.2143 [417.3240 – C_8_H_16_O_2_]^+^ and 255.2040 [273.2143 – H_2_O]^+^ were originated from the consecutive elimination of the E-ring and H_2_O.

Timosaponin G produced a precursor ion [M + Na]^+^ at *m*/*z* 779.4194. It showed a series of fragment ions at *m*/*z* 595.3933 [M + H – 162]^+^, 433.3368 [M + H – 2 × 162]^+^, and 415.3244 [M + H – H_2_O – 2 × 162]^+^, which could be attributed to losses of one glucosyl, one galactosyl, and H_2_O from [M + H]^+^ (*m*/*z* 757.4351). The fragment ions 273.2127 [415.3244 – C_8_H_14_O_2_]^+^ and 255.2000 [273.2127 – H_2_O]^+^ were produced by the consecutive elimination of the E-ring and H_2_O.

*Anemarrhena* saponin I yielded the sodic adduct ion [M + Na]^+^ at *m*/*z* 781.4350, corresponding to its molecular formula as C_39_H_66_O_14_. The main fragment ions at *m*/*z* 741.4505 [M + H – H_2_O]^+^, 579.3817 [M + H – H_2_O – 162]^+^, 417.3410 [M + H – H_2_O – 2 × 162]^+^, and 399.2059 [M + H – 2 × H_2_O – 2 × 162]^+^ were produced directly from the parent ion, which could be attributed to losses of H_2_O, one glucosyl, one galactosyl, and H_2_O. Similarly, the fragment ions at *m*/*z* 271.1636 [417.3410 – C_8_H_18_O_2_]^+^ and 253.1493 [271.1636 – H_2_O]^+^ were also daughter ions produced from the parent ions by consecutive elimination of the E-ring and H_2_O.

Timosaponin AIII displaying a series of fragment ions at *m*/*z* 579.3969 [M + H – 162]^+^, 417.3389 [M + H – 2 × 162]^+^, and 399.3294 [M + H – H_2_O – 2 × 162]^+^ could be attributed to losses of one glucosyl, one galactosyl, and H_2_O from 741.4406 [M + H]^+^. The characteristic fragment ions at *m*/*z* 273.2230 [417.3389 – C_8_H_16_O_2_]^+^ and 255.2105 [273.2230 – H_2_O]^+^ resulted from consecutive losses of E-ring and H_2_O. Timosaponin AIV had the same formula and the similar fragmentation behavior as timosaponin AIII.

**Figure 6:**
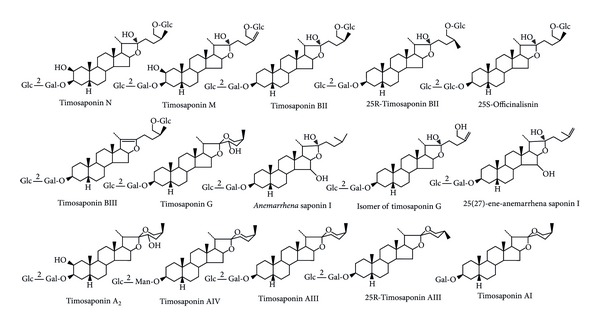
The proposed structures of the fifteen compounds identified by Q-TOF-MS.

**Figure 7:**
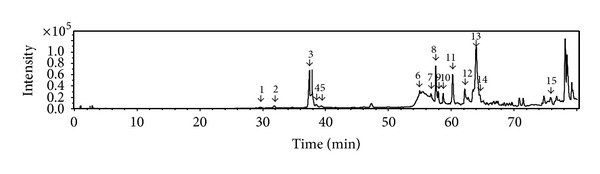
The peak numbers of 60% ethanol fraction.

Based on the exact mass, authentic standards, fragment ions, retention time, and structures in the literature [15–24], the structures of the fifteen compounds (Figure 6) from the 60% ethanol fraction (Figure 7) were deduced as follows.

Peak 1 presented [M + Na]^+^ ion with mass accuracy at *m*/*z* 959.4833. The molecular mass was 16 Da heavier than that of timosaponin BII. It produced fragment ions at  *m*/*z* 919 [M + H – H_2_O]^+^, 757 [M + H – H_2_O – 162]^+^, 595 [M + H – H_2_O – 2 × 162]^+^, 433 [M + H – H_2_O – 3 × 162]^+^, and 415 [M + H – 2 × H_2_O – 3 × 162]^+^, which were attributed to the sequential losses of H_2_O, one galactosyl, two glucosyls, and H_2_O. Moreover, it was noteworthy that the fragment ions at *m*/*z* 271 [415 – C_8_H_16_O_2_]^+^ and 253 [271 – H_2_O]^+^ originating from the consecutive elimination of the E-ring and H_2_O were observed in the MS/MS spectrum. It was suggested that one more hydroxyl group substituent was at C2 position. Based on the fragment ions and retention time, it was tentatively identified as timosaponin N.

Peak 2, the major fragment ions at *m*/*z* 901 [M + H – H_2_O]^+^, 739 [M + H – H_2_O – 162]^+^, 577 [M + H – H_2_O – 2 × 162]^+^, 415 [M + H – H_2_O – 3 × 162]^+^, 273 [415 – C_8_H_14_O_2_]^+^, and 255 [273 – H_2_O]^+^, could be attributed to losses of H_2_O, three hexosyl residues, one formula C_8_H_14_O_2,_ and H_2_O from the ion [M + Na]^+^ (*m*/*z* 941.4726). There was one double bond at the C25 and C27 positions [8]. Peak 2 was identified as timosaponin M.

Peak 3 was definitely identified as timosaponin BII in comparison with an authentic standard. The precursor ion [M + Na]^+^ at  *m*/*z* 943.4886 was the base peak in ESI experiments. Peaks 4 and 5 showed the same formula and similar fragment ions as peak 3. Based on the chromatography behavior of steroidal saponin on a C_18_ column, peak 4 was tentatively identified as the C25R stereoisomer of timosaponin BII. While peak 5 was identified as 25S-officinalisnin-I or its isomer [8].

Peak 6, peak 7, and peak 8 were identified as timosaponin BIII, timosaponin G, and* Anemarrhena* saponin I in comparison with authentic standards.

Peak 9 showed [M + Na]^+^ (*m*/*z* 779.4189) and [M + H – H_2_O]^+^ (*m*/*z* 739.4244) ions. The fragment ions at *m*/*z* 577 [M + H – H_2_O – 162]^+^, 415 [M + H – H_2_O – 2 × 162]^+^, 273 [415 – C_8_H_14_O_2_]^+^, and 255 [273 – H_2_O]^+^ were produced, corresponding to the neutral losses of one glucosyl, one galactosyl, E-ring, and H_2_O. It was tentatively identified as isomer of timosaponin G.

Peak 10 presented [M + Na]^+^ ion with mass accuracy at *m*/*z* 779.4184. In addition to the dehydrated product ion at *m*/*z* 739 [M + H − H_2_O]^+^, a series of characteristic product ions at *m*/*z* 595 [M + H – 162]^+^, 433 [M + H – 2 × 162]^+^, 415 [M + H – H_2_O – 2 × 162]^+^, 271 [415 – C_8_H_16_O_2_]^+^, and 253 [271 – H_2_O]^+^ were also observed. It was tentatively identified as 25(27)-ene-*Anemarrhena* saponin I.

Peak 11 was calculated as C_39_H_64_O_14_ based on the accurate mass measurement [M + Na]^+^ (*m*/*z* 779.4187). The ions at *m*/*z* 757 [M + H]^+^, 595 [M + H – 162]^+^, 433 [M + H – 2 × 162]^+^, 415 [M + H – H_2_O – 2 × 162]^+^, 271 [415 – C_8_H_16_O_2_]^+^, and 253 [271 – H_2_O]^+^ indicated one hydroxyl group substituent of the aglycone. It was tentatively identified as timosaponin A_2_.

Peaks 12 and 13 were identified as timosaponin AIV and timosaponin AIII by authentic standards. Peak 14 yielded the sodic adduct ion [M + Na]^+^ at *m*/*z* 763.4237. It displayed a series of fragment ions at *m*/*z* 579 [M + H – 162]^+^, 417[M + H – 2 × 162]^+^, 273[417 – C8H16O2]^+^, and 255[273 – H2O]^+^. The retention time of steroidal saponins with the 25R configuration was longer than those with the 25S configuration. Peak 14 was tentatively identified as 25R-timosaponin AIII.

Peak 15 showed the [M + H]^+^ ion at *m*/*z* 579.3892 (C_33_H_55_O_8_), 162 Da less than that of timosaponin AIII. The product ions such as *m*/*z* 417 [M + H – 162]^+^, 399 [M + H – H_2_O – 162]^+^, 273 [417 – C_8_H_16_O_2_]^+^, and 255 [273 – H_2_O]^+^ could be found in their MS/MS spectrum. From this, peak 15 was identified as timosaponin A-I.

### 3.4. Isolation and Purification of Compounds from* Anemarrhena asphodeloides* Bge

**Figure 8:**
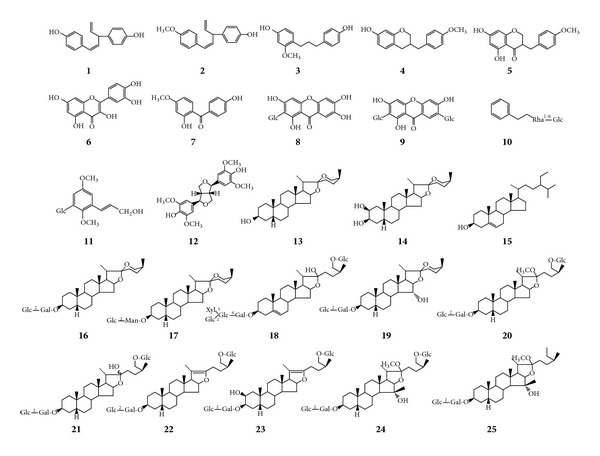
The structures of the isolated compounds.

Twenty-five compounds were obtained after repeated isolation and purification. On the basis of spectroscopic analysis (MS, ^1^H NMR, ^13^C NMR, DEPT135, HSQC, HMBC, and NOESY spectra) and in comparison with the previously reported spectral data [25–34], the structures of these compounds were shown in Figure 8. All the isolates were subjected to the cholinesterase inhibitory activity assay (Table 3).

## 4. Discussion

The anticholinesterase activity of the agents was tested in the presence of a known concentration. It was one of the approaches to study the effect of treating Alzheimer's disease. The anticholinesterase inhibition ratio of the agents was expressed as percentage inhibition ratio based on the optical density. The conditions of AChE and BuChE inhibitory assay were confirmed by a series of literature reports and experiments.

To determine which fraction has anticholinesterase activity, the rhizome of* Anemarrhena asphodeloides* was fractionated further by a series of extractions with different solvents and techniques. The findings revealed that more bioactive components would be extracted by dichloromethane, ethyl acetate, and acetone. The extracts dealt with by the ultrasonic and cold soak method had higher inhibition ratio than the extracts dealt with by the heat reflux method. It is proposed that the molecular structures of the active constituents may be destroyed at a high temperature.

The effect of ethyl acetate extract extracted by the ultrasonic method on inhibiting activity of cholinesterase was better than those dealt with by other solvents and methods. The ethyl acetate crude extract of* Anemarrhena asphodeloides* may also contain many compounds with differing polarities, so it was fractionated further by a D-101 macroporous resin column. The bioactive compounds of the 60% ethanol fraction were analyzed by HPLC-QTOF-MS system.

The type of compounds in the 60% ethanol fraction could be deduced by the MS data. If the ions [M + Na]^+^ and [M + H − H_2_O]^+^ were detected and the [M+H]^+^ ion was not observed, the saponin would be a furostanol saponin with the C22 hydroxyl group. If the ions at *m*/*z* 417, 255, and 273 were detected, there should be no substituent attached to the sarsasaponin. If the ions at *m*/*z* 433 and 415 were observed, there should be one more hydroxyl group substituent on the aglycone. The retention time of saponins would be affected by the number, classes, and connection orders of sugar moieties and the number and position of hydroxyl group substituent at the aglycone. What is more, the retention time of steroidal saponins with the 25R configuration was longer than those with the 25S configuration on a C18 column.

Twenty-five compounds were isolated from the active fraction. The structure-activity relationships were discussed based on the results of cholinesterase inhibitory activity. It showed that the compounds with the C_6_–C_3_–C_6_ skeleton probably had both AChE and BuChE inhibitory activities. Compound** 3** showed potential BuChE inhibitory activitity with the inhibition ratio of 75.8%. Xanthone and benzene derivatives exhibited no or little activity. Lignans showed weak BuChE inhibitory activity. The steroidal saponins had moderate or weak AChE inhibitory activity. It was supposed that adding OH groups to the steroidal saponins may increase the effect a little. Although some of the individual compounds exhibited potential* in vitro* activity, they were less effective than the 60% ethanol fraction and this might be due to a synergistic action among the active components or maybe the most active constituents have not been isolated by us.

Steroid saponins were considered as potentially drug preventing and treating neurodegenerative diseases, such as Alzheimer's disease. However, we found that compounds with the C_6_–C_3_–C_6_ skeleton probably had both AChE and BuChE inhibitory activities, especially compound** 3**. The activity* in vivo* needs to be tested in the future.

## 5. Conclusion

AChE and BuChE inhibitory activities of* Anemarrhena asphodeloides* Bge extracted with three different techniques and ten kinds of solvents were studied.* Anemarrhena asphodeloides* Bge extracted with ethyl acetate by the ultrasonic technique had the most potential anticholinesterase activity. The extract was partitioned into six fractions after applying to a D-101 macroporous resin column, and active compounds were concentrated into 60% ethanol fraction. Fifteen main peaks of this fraction were identified and tentatively characterized based on the HPLC-QTOF-MS technique, standards, and literature reports. Twenty-five compounds were obtained from 60% ethanol fraction after repeated isolation and purification. The structure-activity relationships were discussed based on the results of cholinesterase inhibitory activity.

## Supplementary Material

Twenty-five compounds were obtained after repeated isolation and purification from the active fraction. Their structures were established by detailed spectral studies and by the comparison with literature data. The main spectrum of the isolated compounds were exhibited in the supplementary material.

## Figures and Tables

**Table 1:** The effects of the tested extracts on AChE and BuChE inhibitory activities.

Solvents	AChE inhibition (%)			BuChE inhibition (%)		
	Ultrasonic method	Heat reflux method	Cold soak method	Ultrasonic method	Heat reflux method	Cold soak method
Petroleum ether	—	—	—	—	—	1.6 ± 0.1
Dichloromethane	—	—	—	41.5 ± 0.5	15.7 ± 0.7	41.2 ± 0.6
Ethyl acetate	—	—	—	44.1 ± 0.5	27.7 ± 0.4	40.8 ± 0.8
Acetone	—	—	—	38.8 ± 0.3	22.2 ± 0.3	37.9 ± 0.7
Methanol	—	—	—	12.3 ± 0.4	5.4 ± 0.2	7.1 ± 0.3
95% ethanol	—	—	—	4.9 ± 0.2	2.8 ± 0.1	3.9 ± 0.3
70% ethanol	—	—	—	1.8 ± 0.1	—	1.6 ± 0.2
50% ethanol	—	—	—	2.8 ± 0.1	—	0.5 ± 0.1
30% ethanol	—	—	—	2.4 ± 0.1	—	1.8 ± 0.2
Water	—	—	—	—	—	—

AChE inhibition ratio of donepezil: 98.0 ± 0.5; BuChE inhibition ratio of donepezil: 80.2 ± 0.6.

**Table 2:** HPLC/Q-TOF MS analysis for accurate mass measurements of constituents in 60% ethanol fraction.

Number	*T*_*R*_ (min)	Formula	Selected ion	Theoretical mass (*m*/*z*)	Experimental mass (*m*/*z*)	Error (ppm)	Fragment ions in positive (+) ion mode	Identification
1	29.8	C_45_H_76_O_20_	[M + Na]^+^	959.4822	959.4833	−1.1	919, 757, 595, 433, 415, 271, 253	Timosaponin N
2	31.7	C_45_H_74_O_19_	[M + Na]^+^	941.4717	941.4726	−1.0	901, 739, 577, 415, 273, 255	Timosaponin M
3	37.6	C_45_H_76_O_19_	[M + Na]^+^	943.4873	943.4886	−1.4	903, 741, 579, 417, 273, 255	Timosaponin BII
4	38.5	C_45_H_76_O_19_	[M + Na]^+^	943.4873	943.4882	−1.0	903, 741, 579, 417, 273, 255	25R-Timosaponin BII
5	39.2	C_45_H_76_O_19_	[M + Na]^+^	943.4873	943.4871	0.2	903, 741, 579, 417, 273, 255	25S-Officinalisnin-I
6	54.9	C_45_H_74_O_18_	[M + Na]^+^	925.4767	925.4788	−2.3	741, 579, 417, 273, 255	Timosaponin BIII
7	56.7	C_39_H_64_O_14_	[M + Na]^+^	779.4188	779.4202	−1.8	739, 595, 433, 415, 273, 255	Timosaponin G
8	57.6	C_39_H_66_O_14_	[M + Na]^+^	781.4345	781.4367	−2.8	741, 579, 417, 399, 271, 253	*Anemarrhena* saponin I
9	58.0	C_39_H_64_O_14_	[M + Na]^+^	779.4188	779.4189	−0.1	739, 577, 415, 273, 255	Isomer of timosaponin G
10	58.6	C_39_H_64_O_14_	[M + Na]^+^	779.4188	779.4184	0.5	739, 595, 433, 415, 271, 253	25(27)-ene-*Anemarrhena* saponin I
11	60.4	C_39_H_64_O_14_	[M + Na]^+^	779.4188	779.4187	0.1	757, 595, 433, 415, 271, 253	Timosaponin A_2_
12	62.2	C_39_H_64_O_13_	[M + Na]^+^	763.4239	763.4237	0.3	579, 417, 273, 255	Timosaponin AIV
13	63.9	C_39_H_64_O_13_	[M + Na]^+^	763.4239	763.4241	−0.3	579, 417, 273, 255	Timosaponin AIII
14	64.4	C_39_H_64_O_13_	[M + Na]^+^	763.4239	763.4221	2.4	579, 417, 273, 255	25R-Timosaponin AIII
15	75.8	C_33_H_54_O_8_	[M + Na]^+^	601.3711	601.3703	1.3	417, 273, 255	Timosaponin AI

**Table 3:** The effects of the twenty-five compounds on AChE and BuChE inhibitory activities.

Compounds	AChE inhibition (%)	BuChE inhibition (%)
**1**	17.9 ± 0.3	37.6 ± 0.6
**2**	15.2 ± 0.2	28.1 ± 0.4
**3**	12.5 ± 0.4	75.8 ± 0.7
**4**	—	—
**5**	14.1 ± 0.3	35.3 ± 0.5
**6**	17.9 ± 0.2	36.0 ± 0.4
**7**	14.2 ± 0.5	0.6 ± 0.1
**8**	—	—
**9**	—	—
**10**	0.9 ± 0.1	0.1 ± 0.1
**11**	7.9 ± 0.3	4.0 ± 0.2
**12**	1.7 ± 0.2	15.1 ± 0.3
**13**	18.5 ± 0.4	0.5 ± 0.1
**14**	27.2 ± 0.2	4.2 ± 0.2
**15**	21.6 ± 0.4	—
**16**	1.1 ± 0.1	—
**17**	13.2 ± 0.3	—
**18**	15.9 ± 0.4	—
**19**	23.5 ± 0.4	12.3 ± 0.6
**20**	18.4 ± 0.4	—
**21**	19.2 ± 0.3	—
**22**	17.3 ± 0.3	—
**23**	20.3 ± 0.5	7.3 ± 0.2
**24**	21.1 ± 0.6	—
**25**	22.4 ± 0.2	2.8 ± 0.2
Donepezil	98.2 ± 0.8	79.8 ± 0.7
