# Supplementary material for: A Platform for Screening Potential Anticholinesterase Fractions and Components Obtained from Anemarrhena asphodeloides Bge for Treating Alzheimer's Disease
Source: Evid Based Complement Alternat Med. 2014 Apr 17;2014:524650. doi: 10.1155/2014/524650 (PMC4016847; doi:10.1155/2014/524650)

**Supplementary Materials: The main spectrum of the isolated compounds.**

**Compound 1:**

**
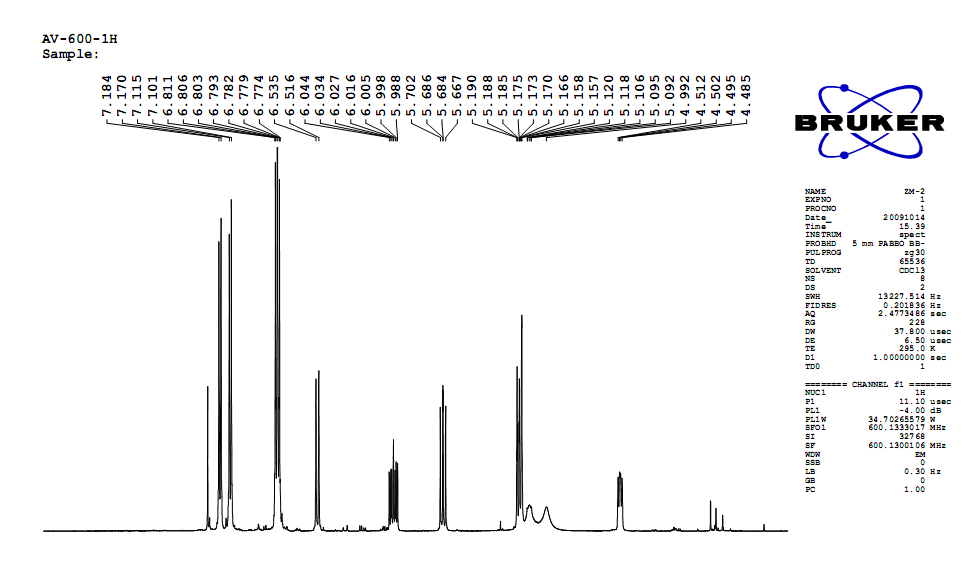
**


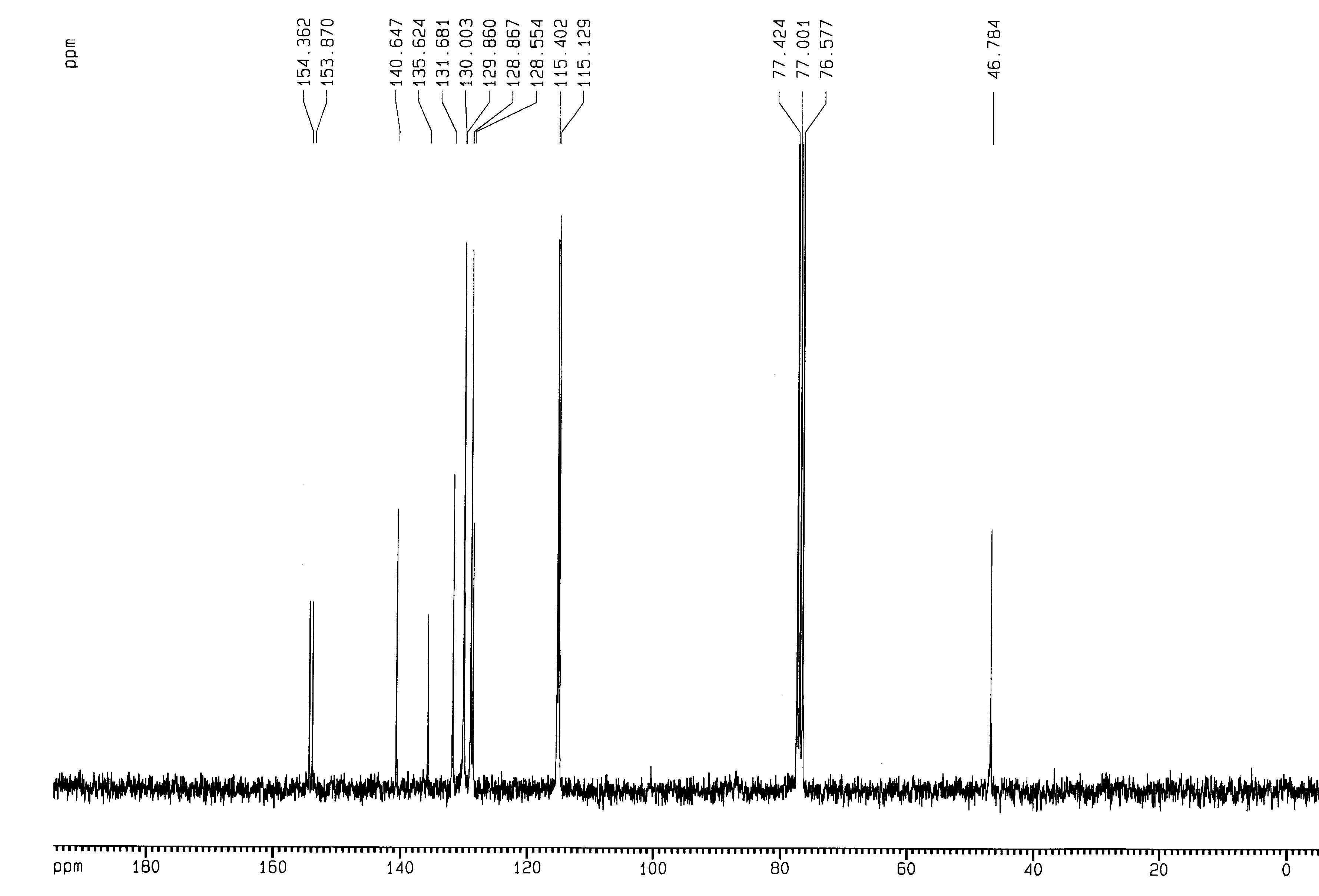


**Compound 2:**


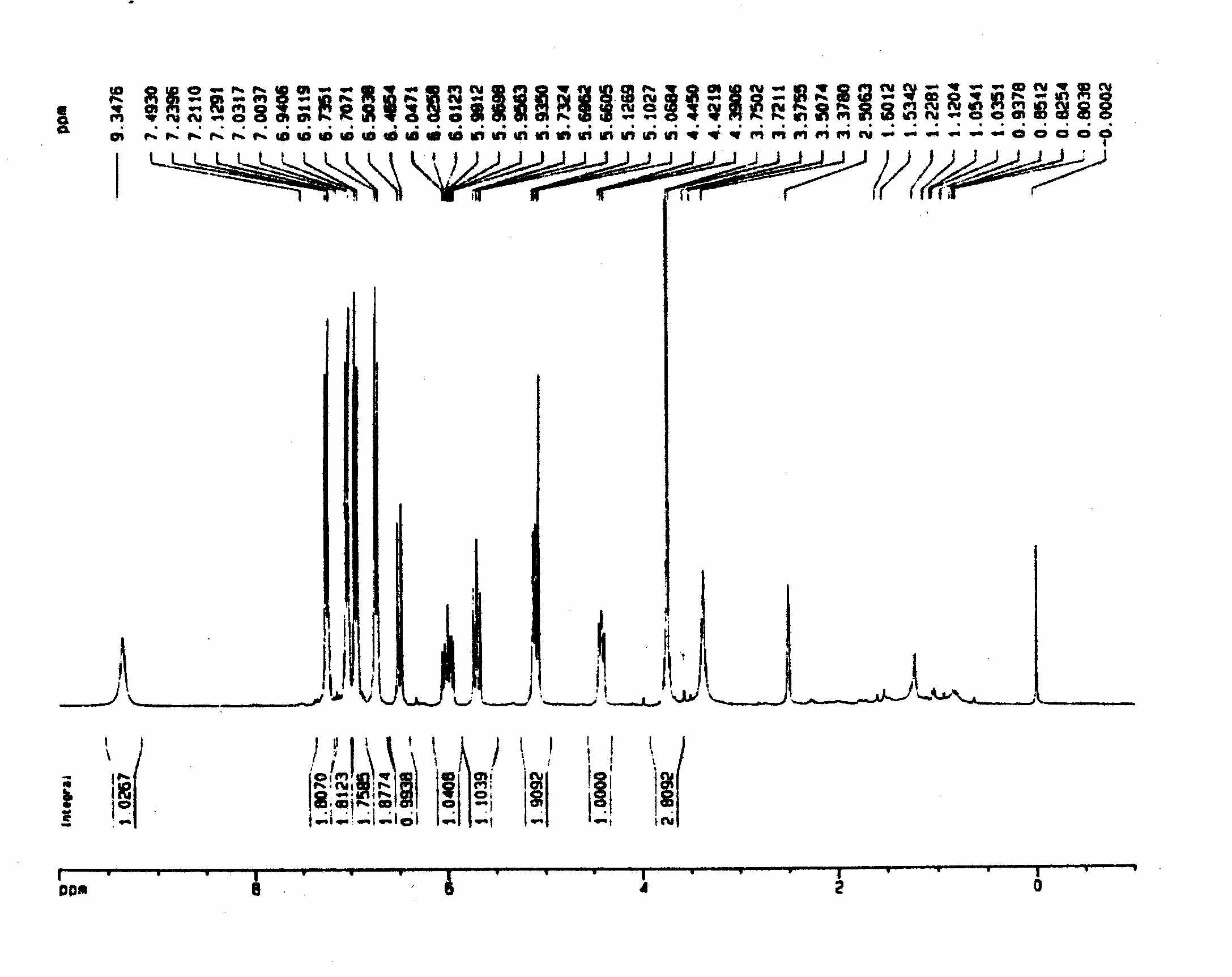

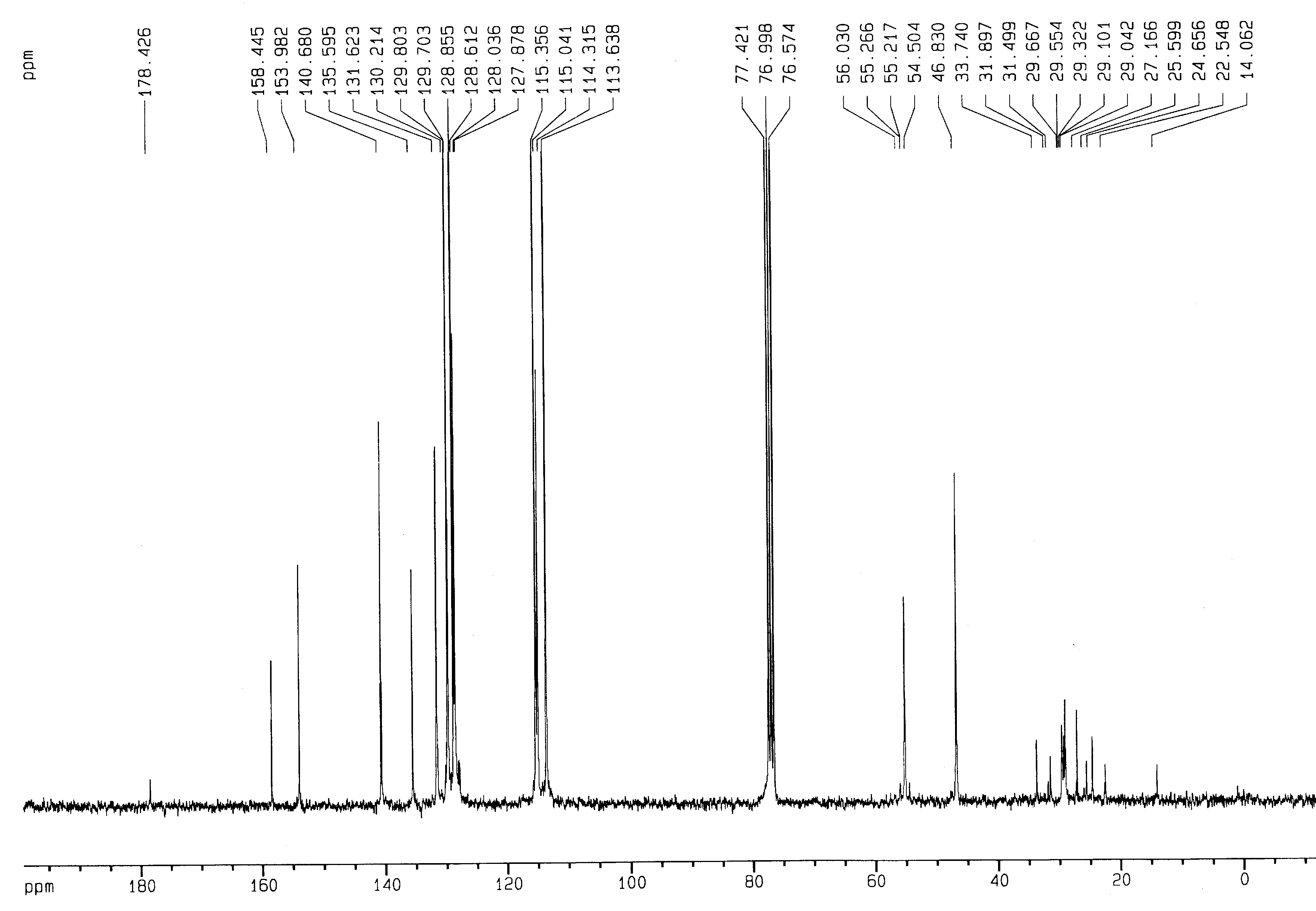


**Compound 3:**

**
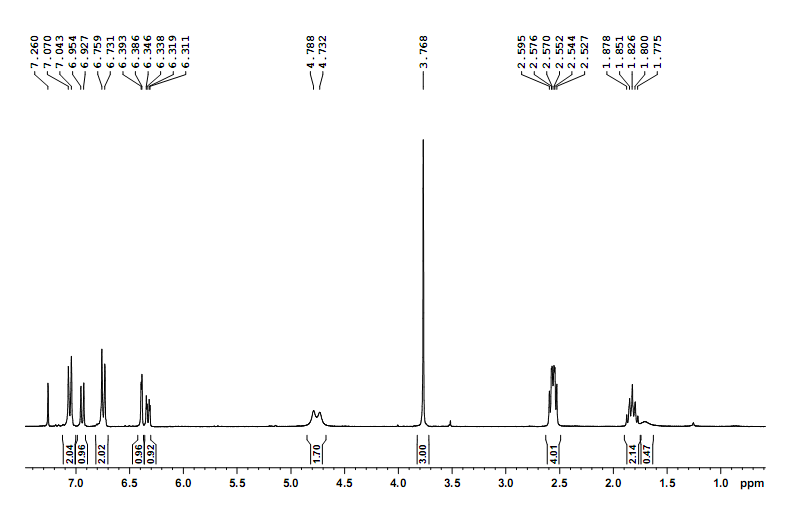
**

**Compound 4:**

**
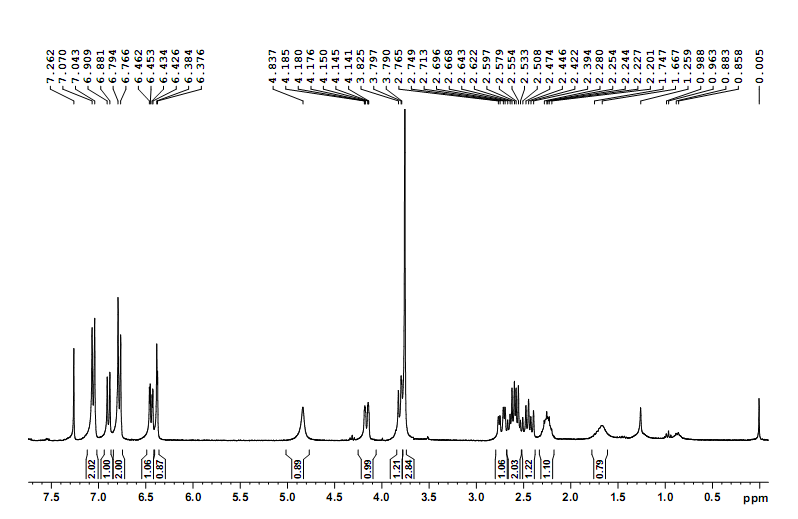

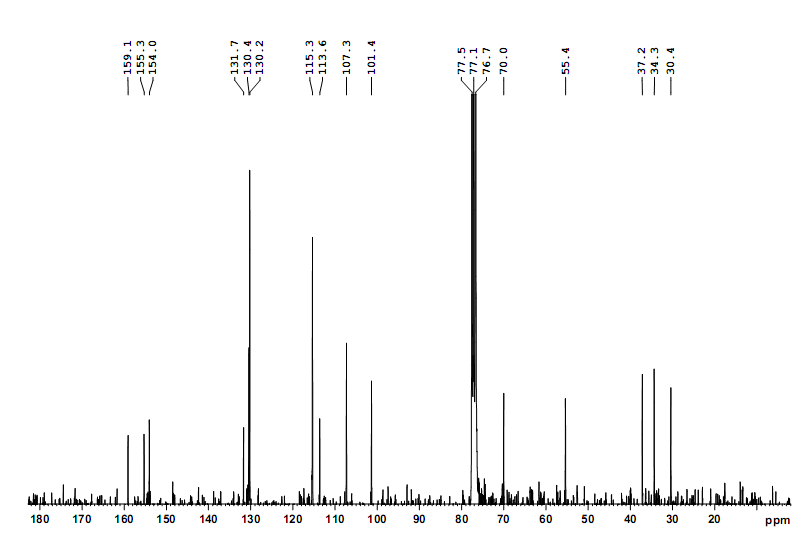
**

**Compound 5:**


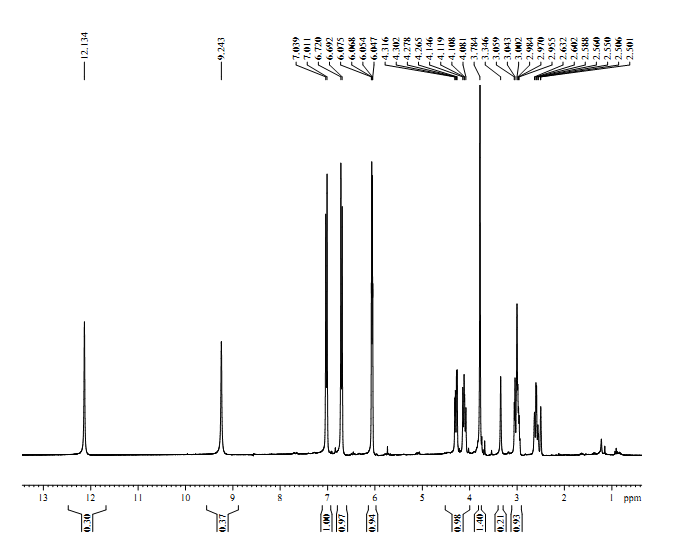

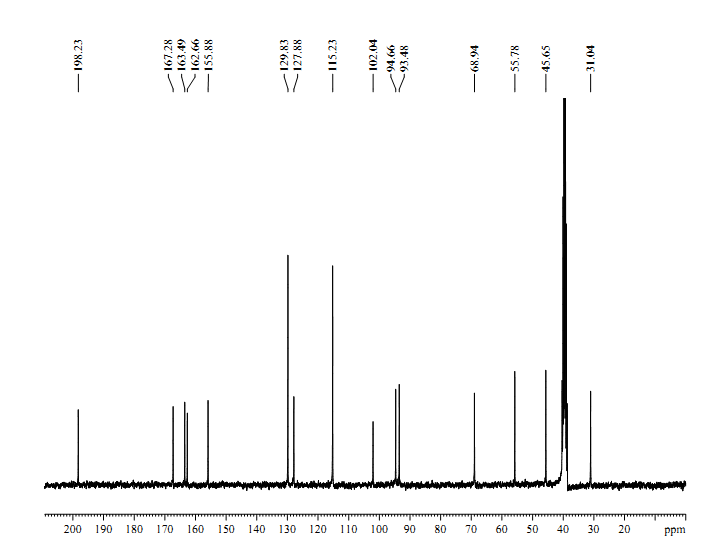


**Compound 6:**


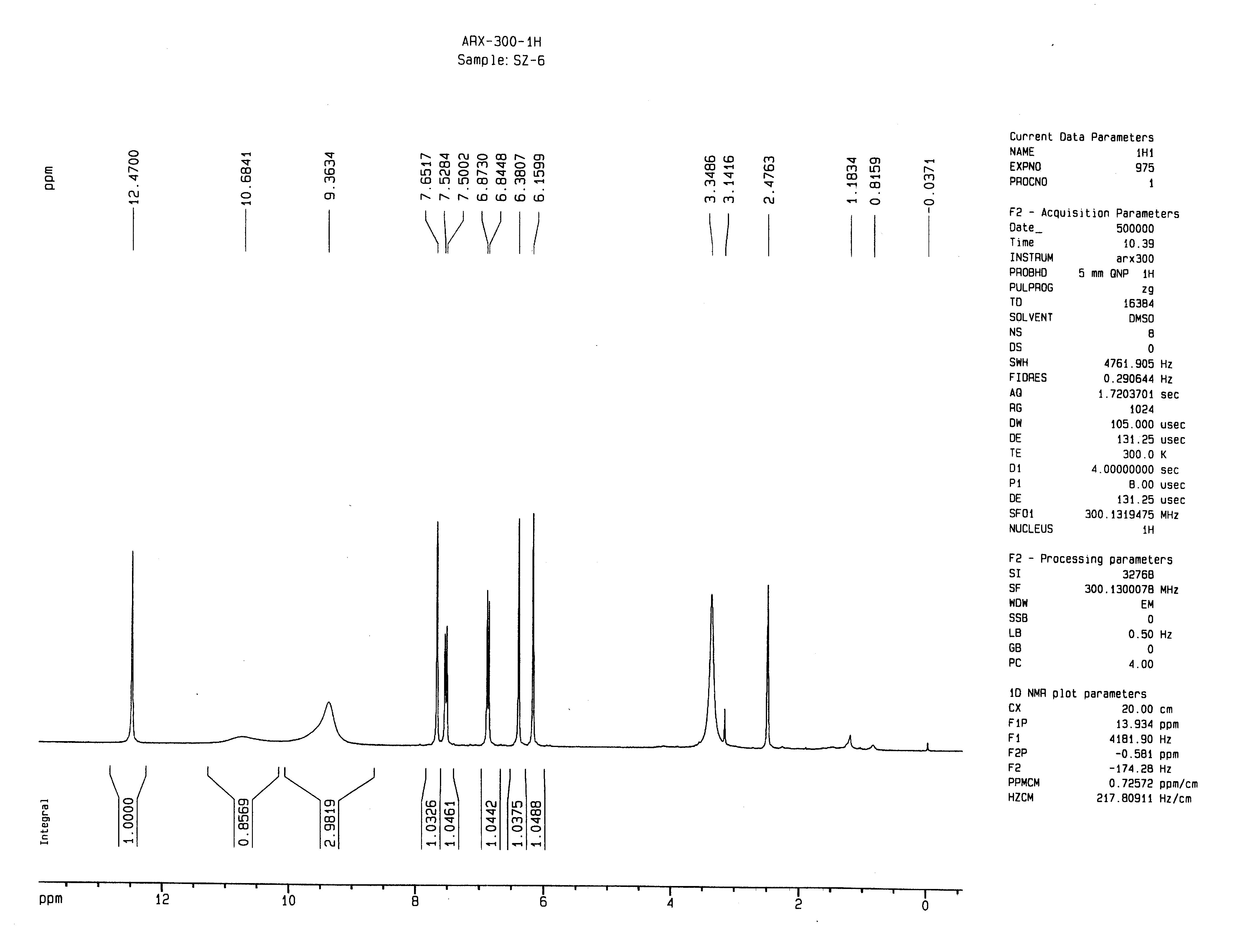

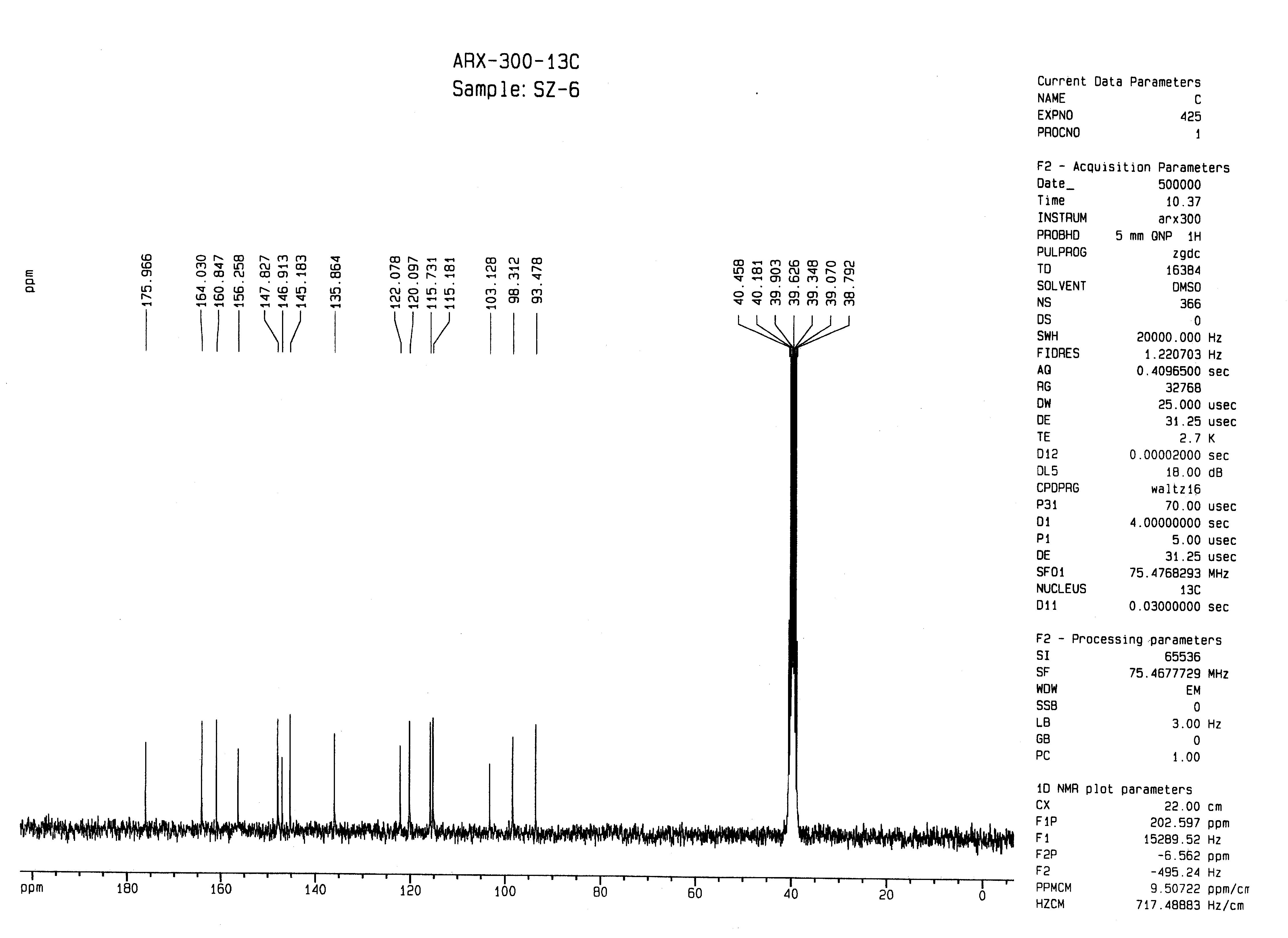


**Compound 7:**


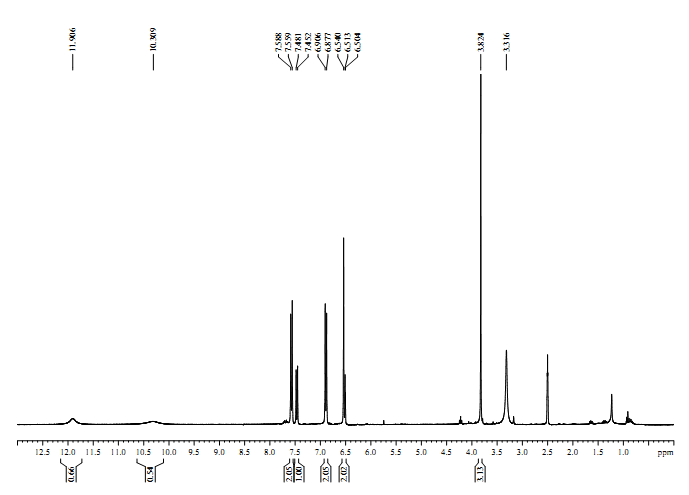

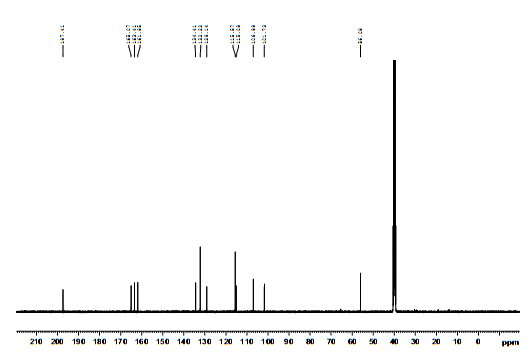


**Compound 8:**


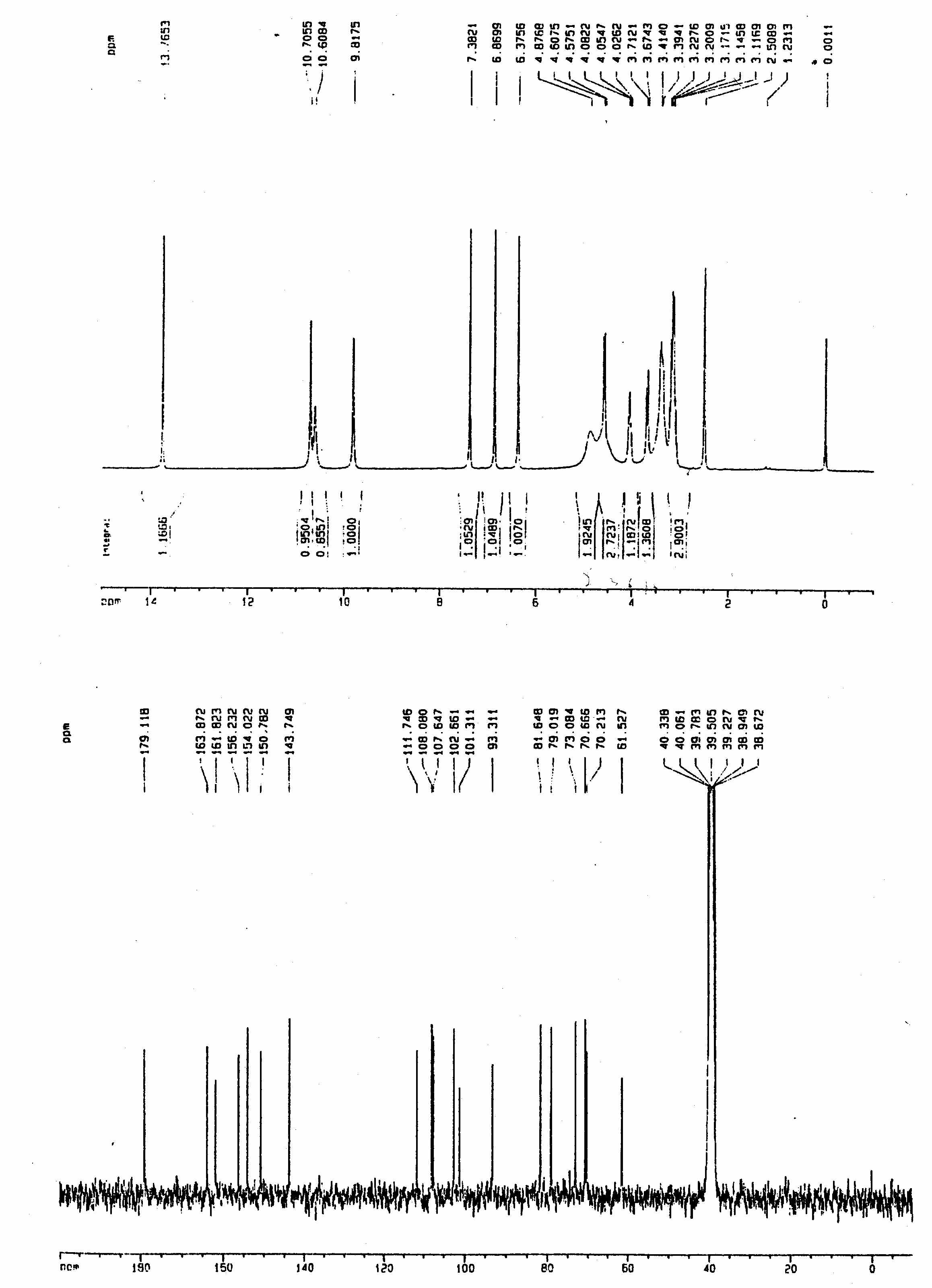

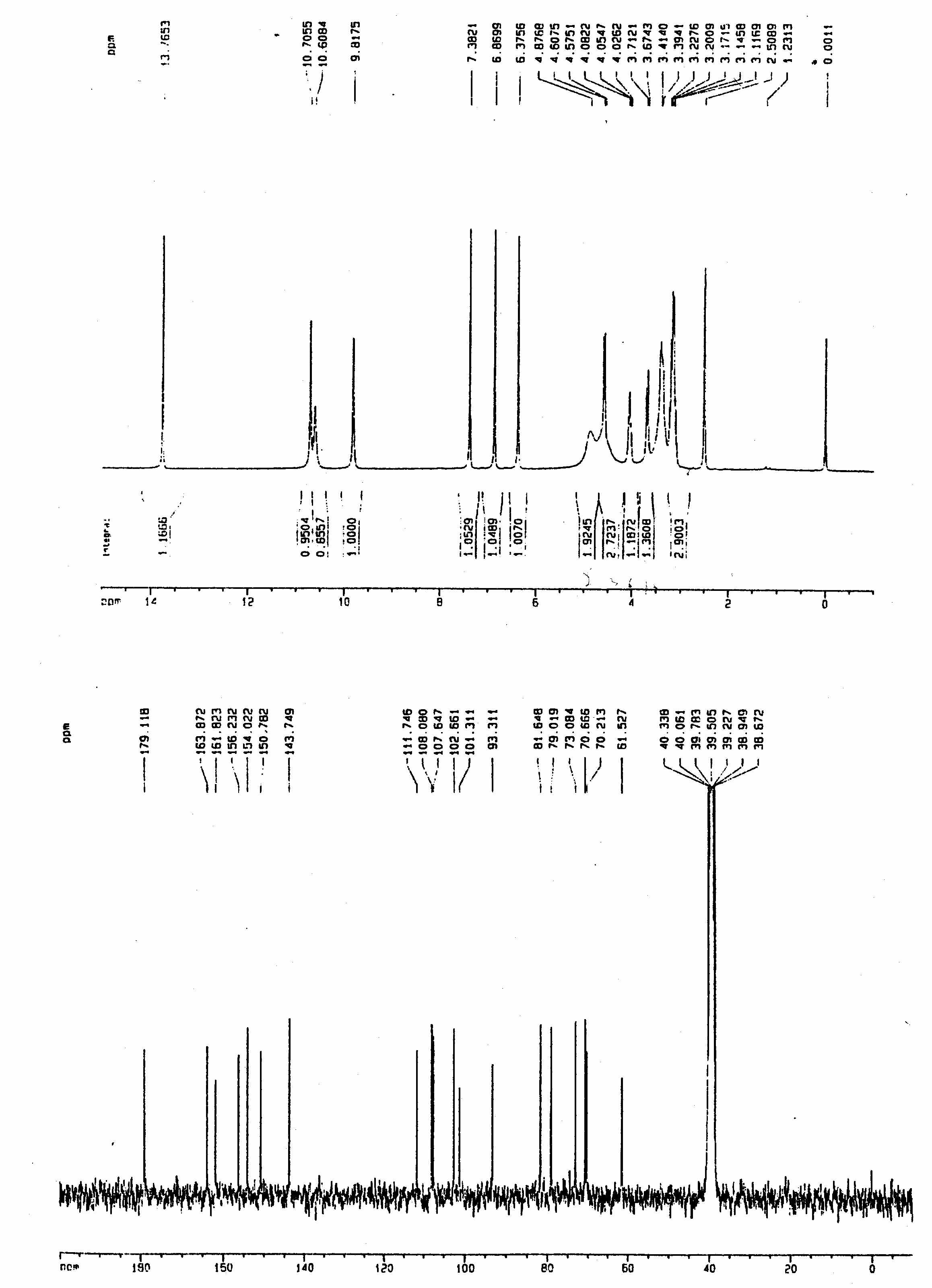


**Compound 9:**


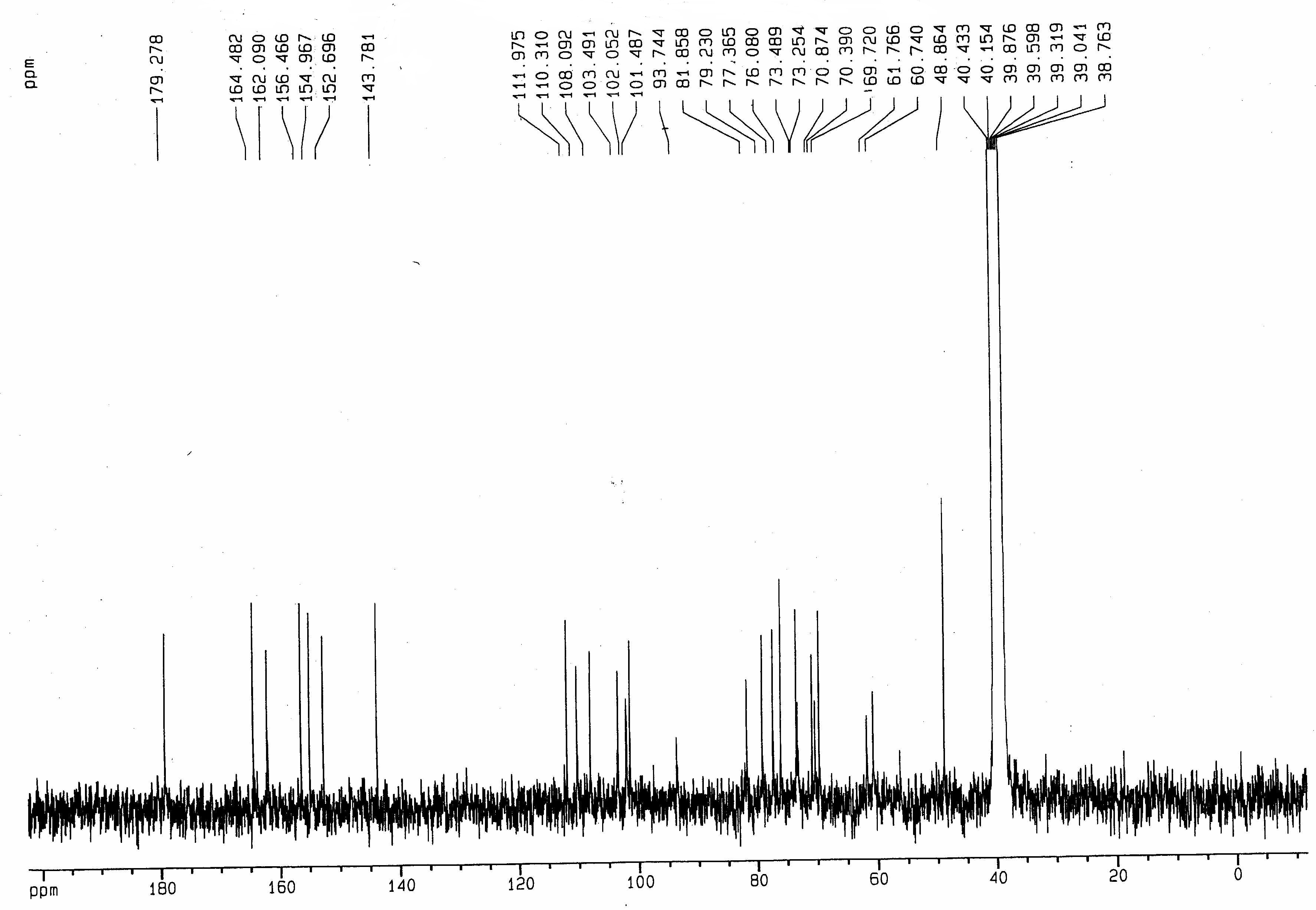


**Compound 10:**


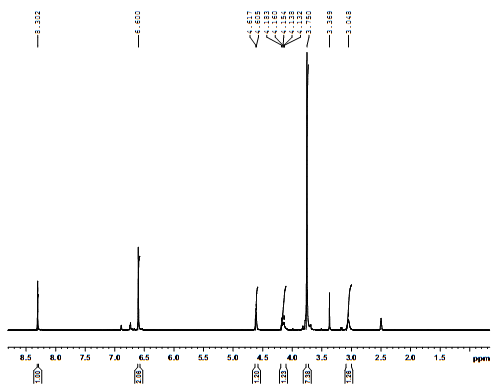

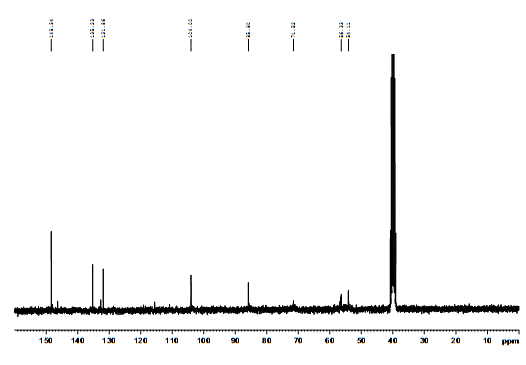


**Compound 11:**


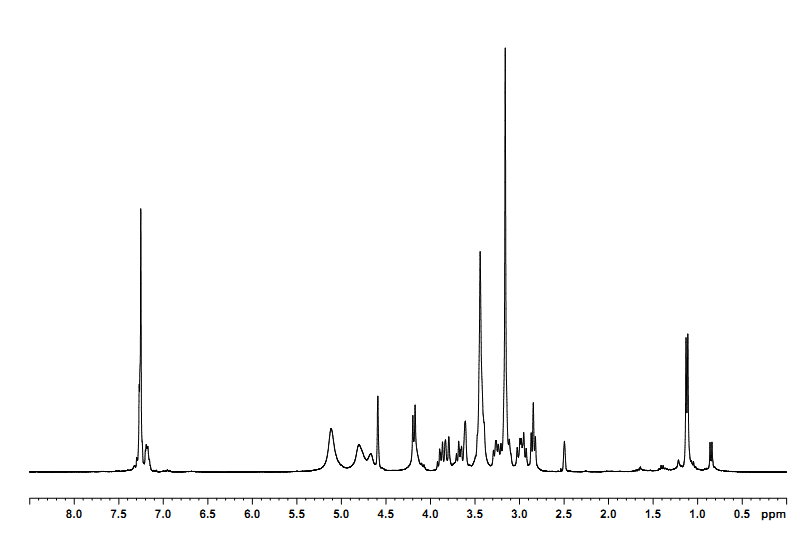

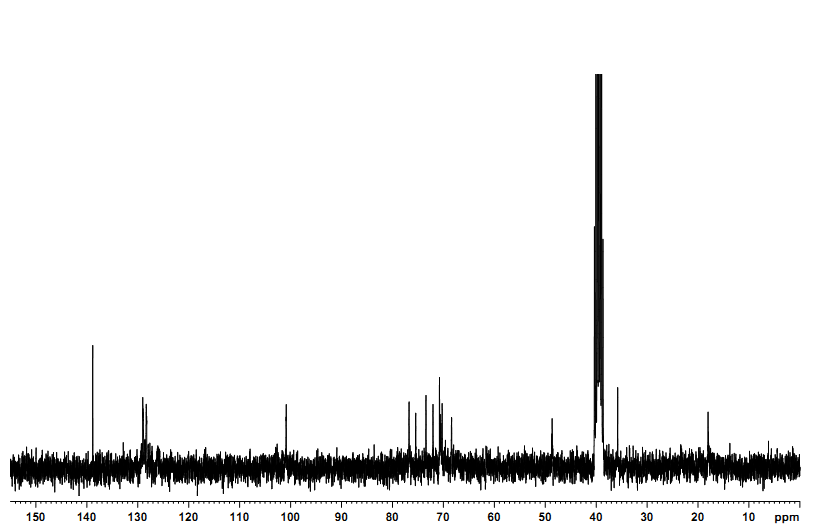


**Compound 12:**


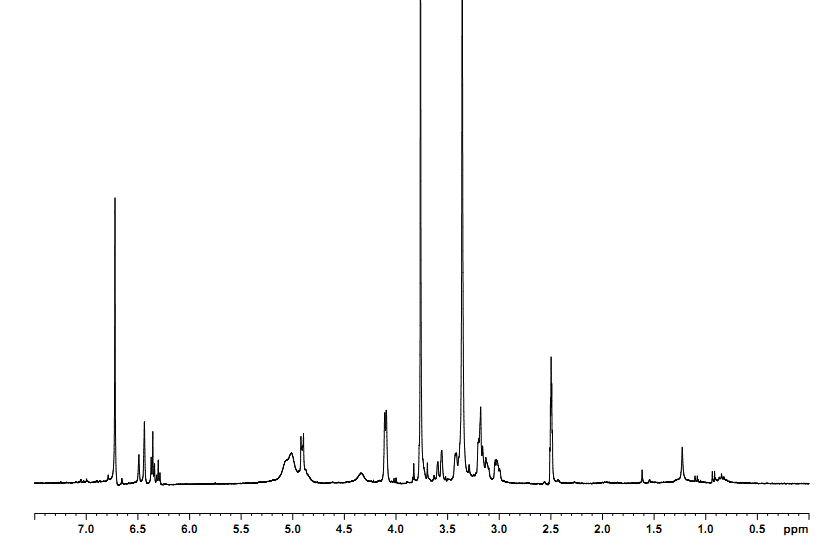

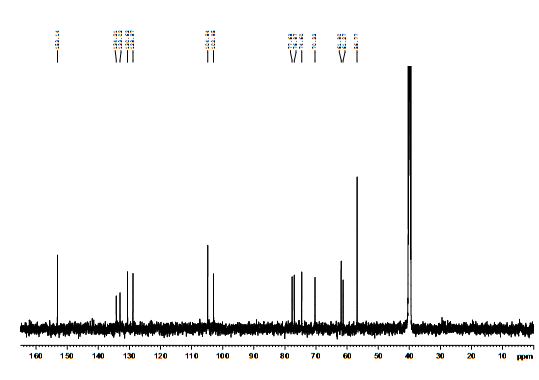


**Compound 13:**


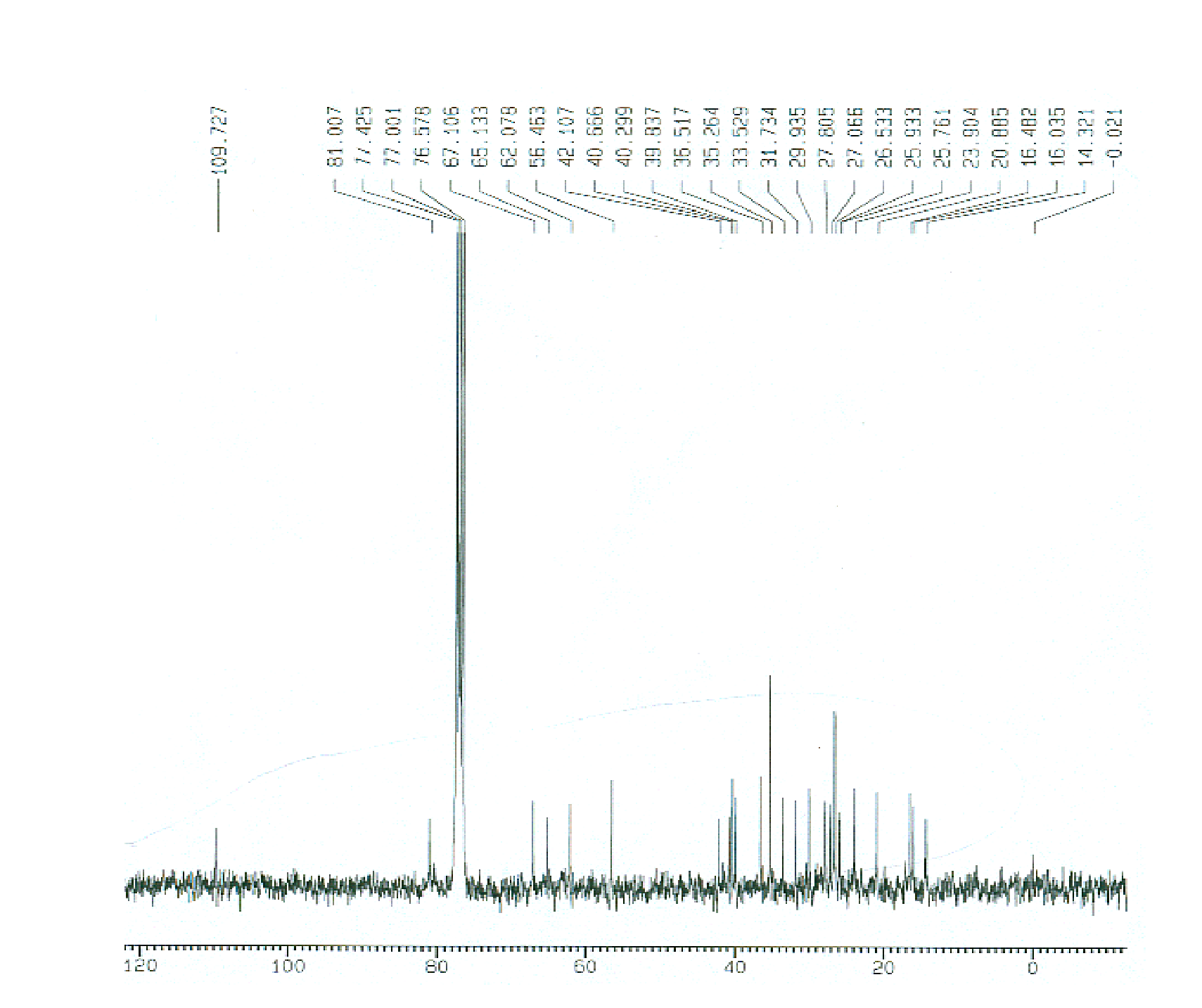


**Compound 14:**


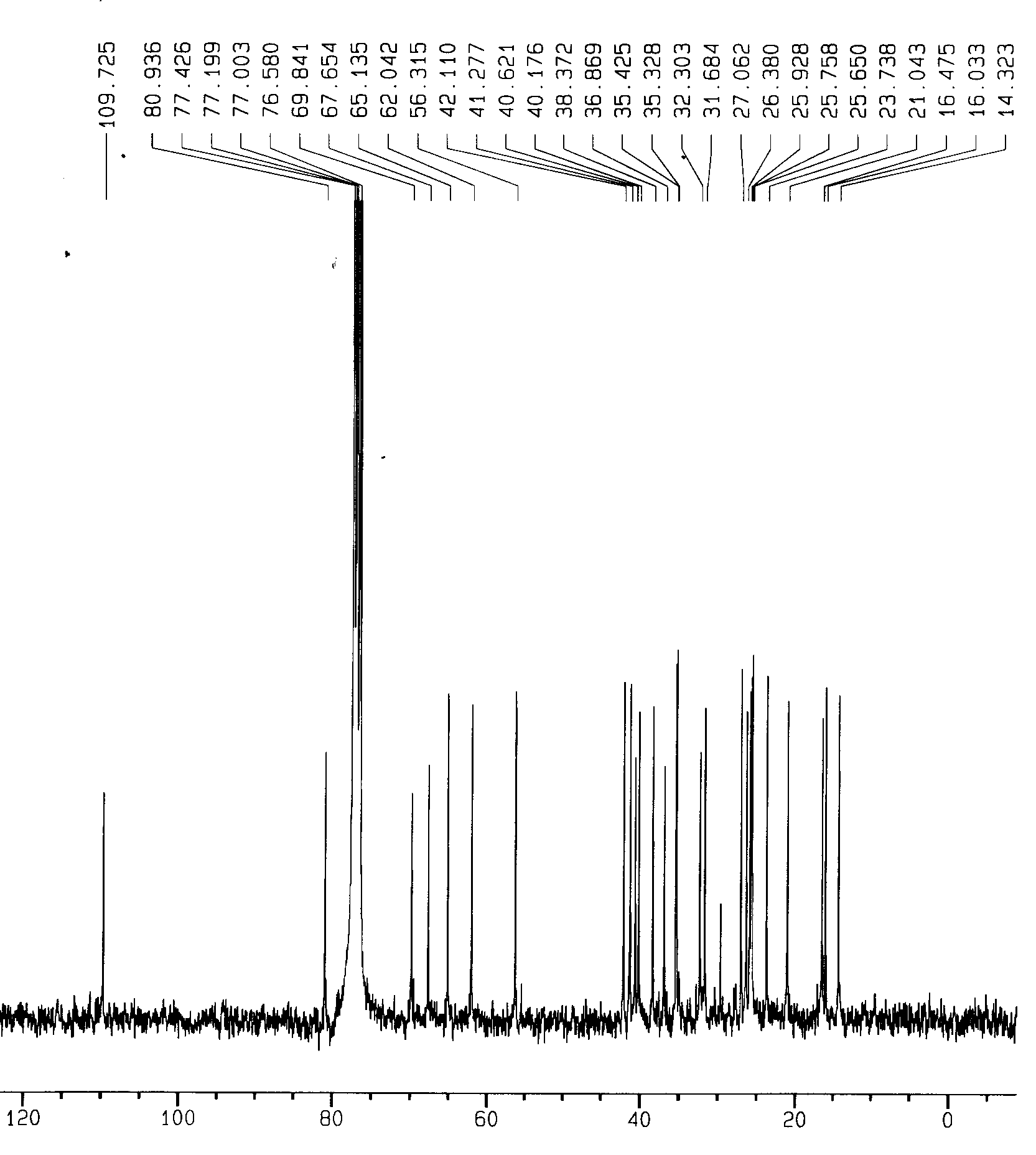


**Compound 15:**

**
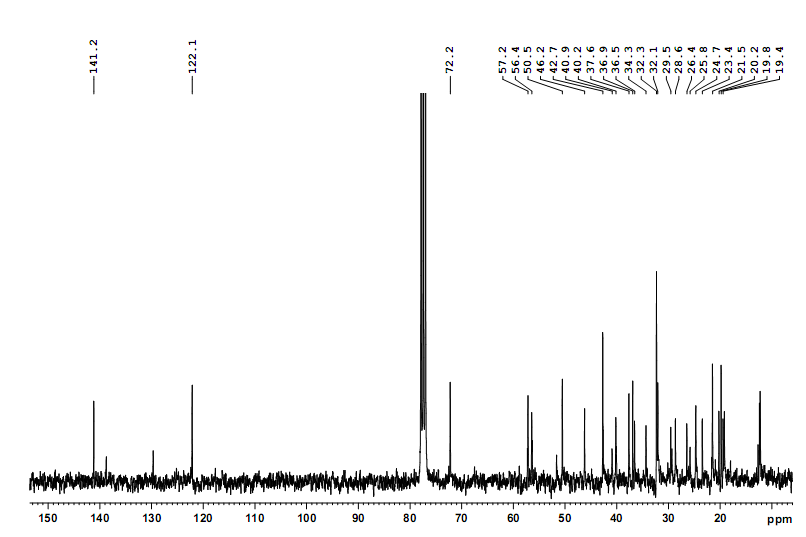
**

**Compound 16:**


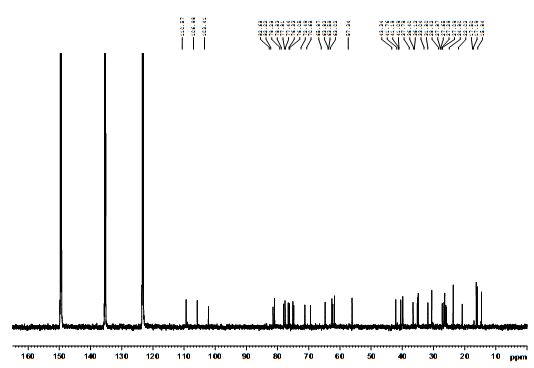


**Compound 17:**


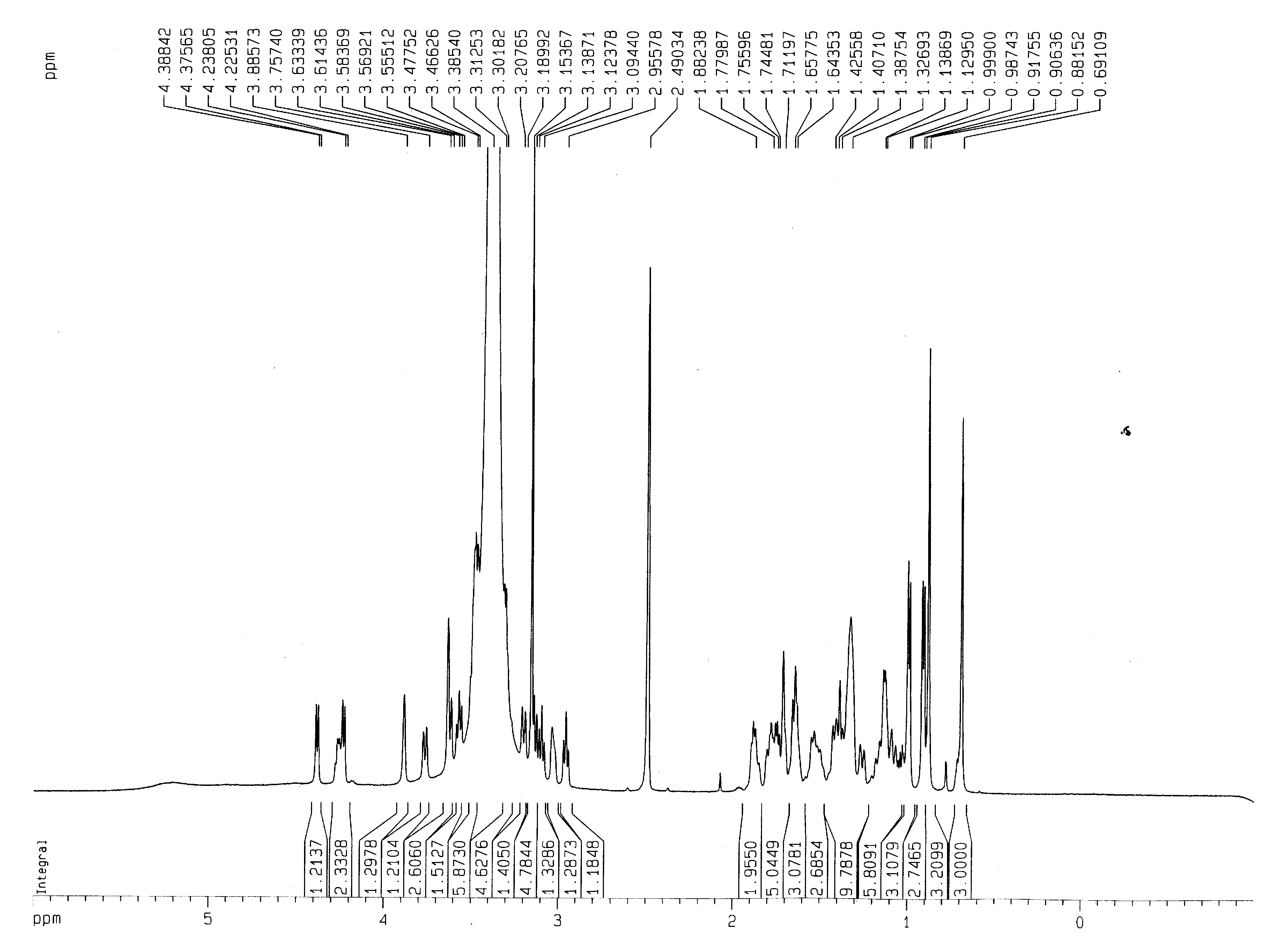


**Compound 17:**


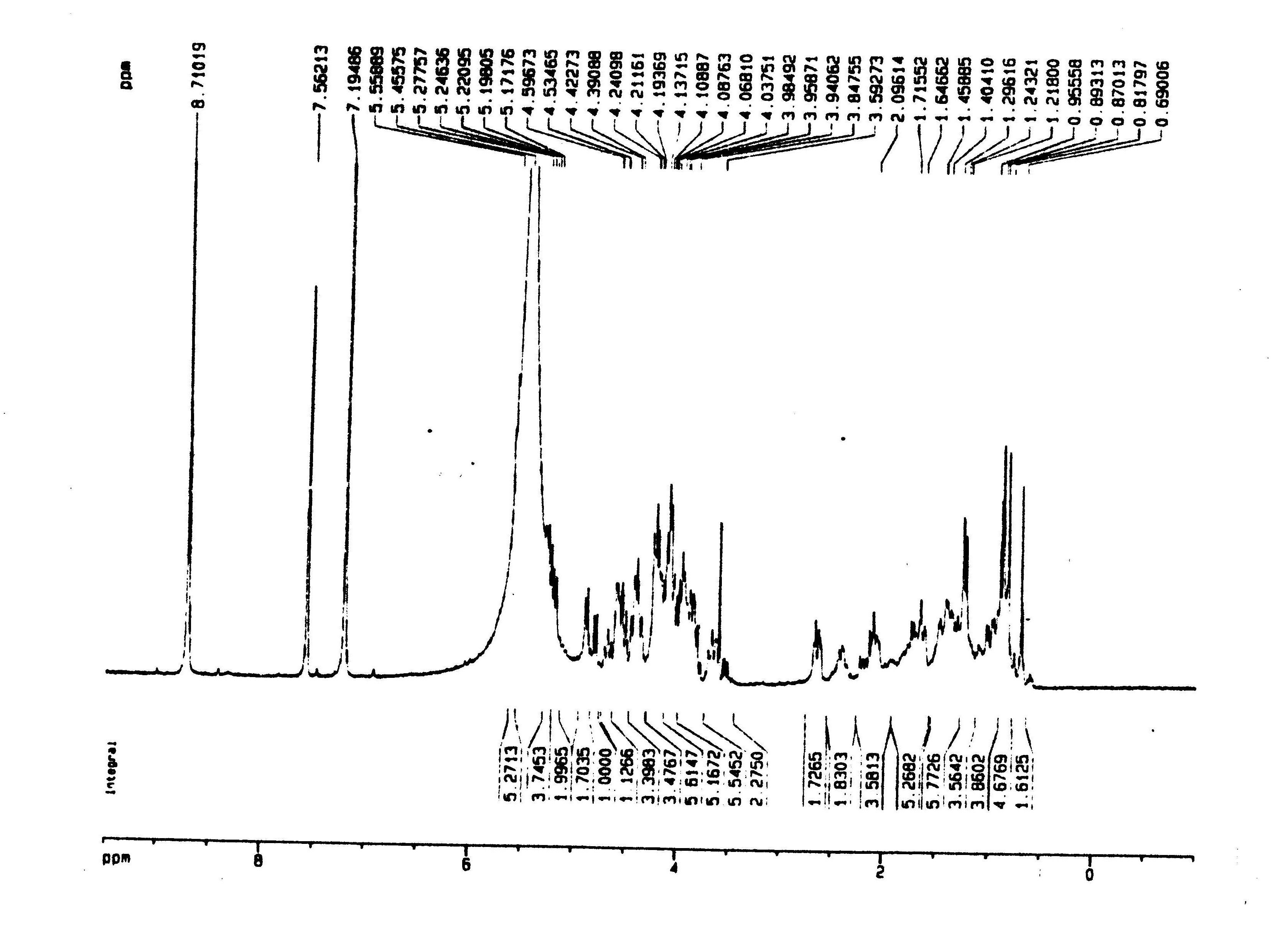

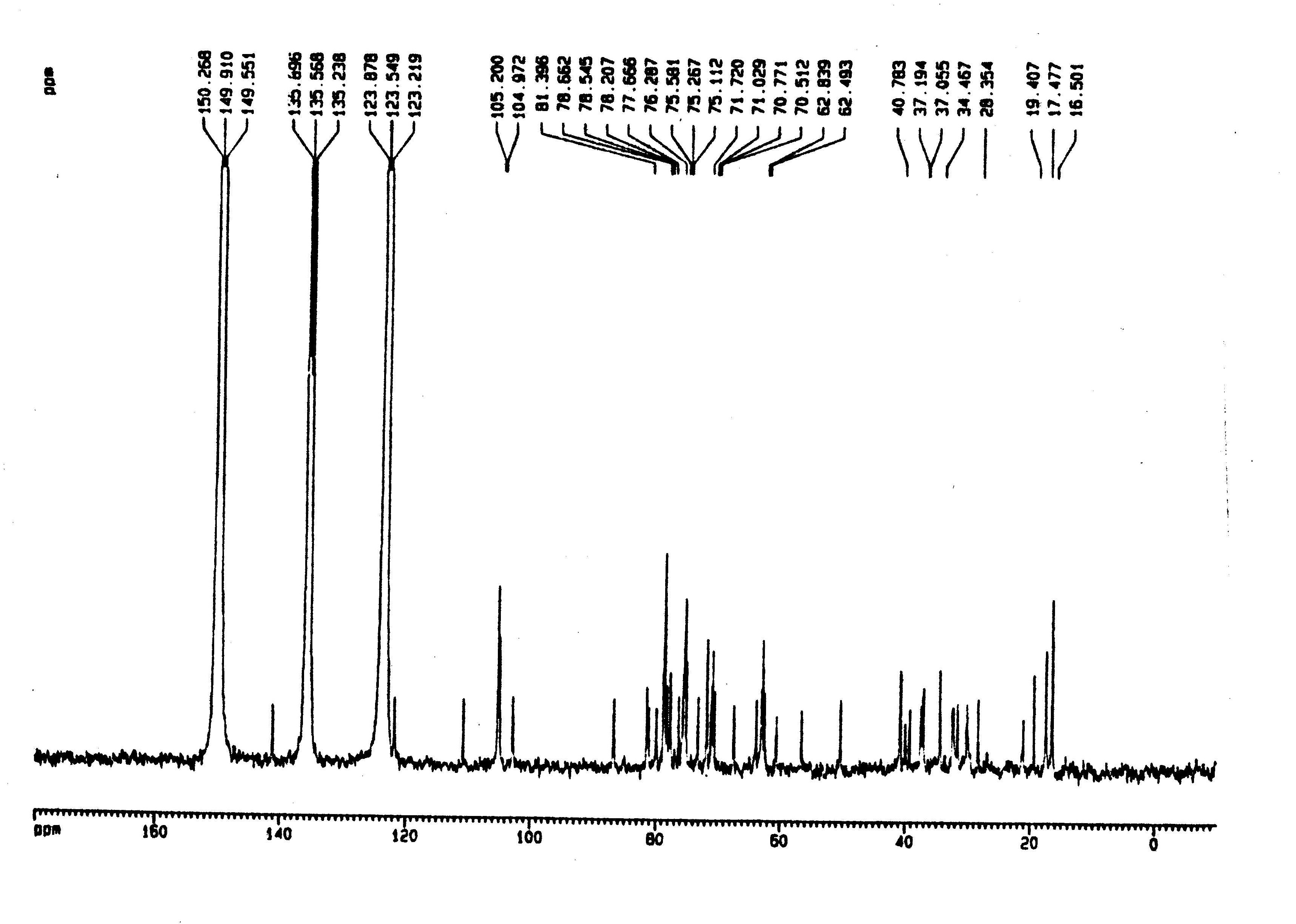


**Compound 18:**


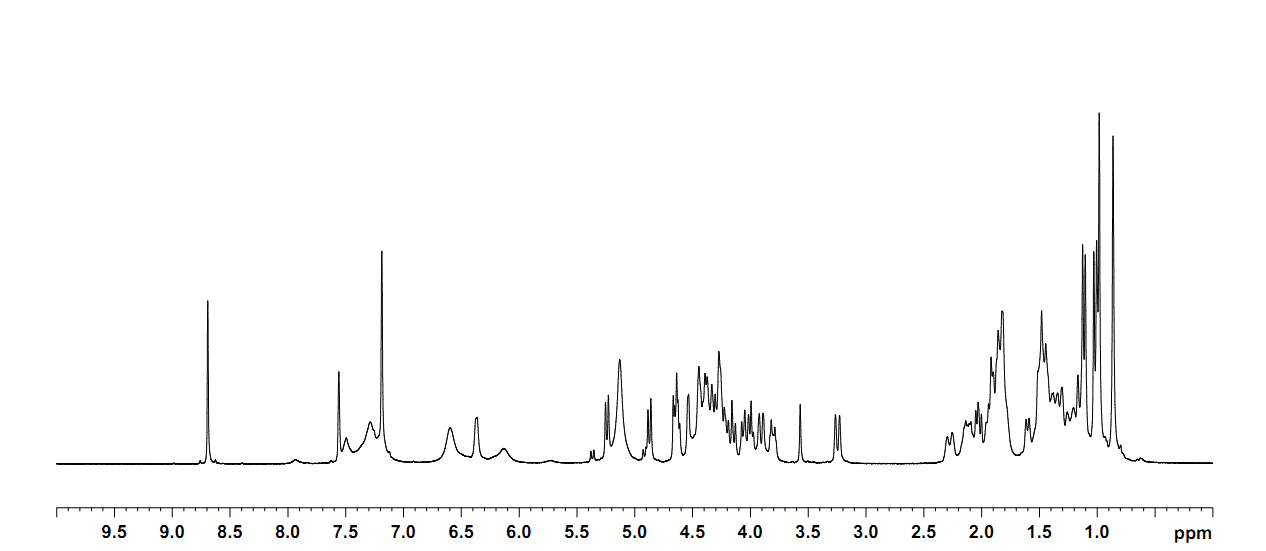

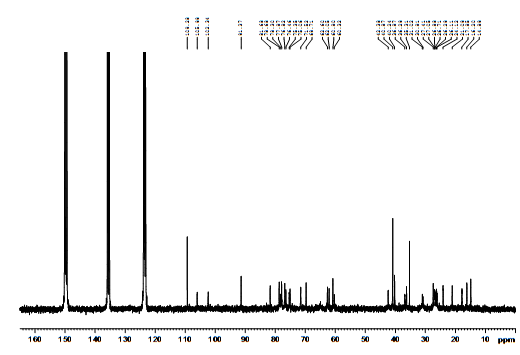


**Compound 19:**


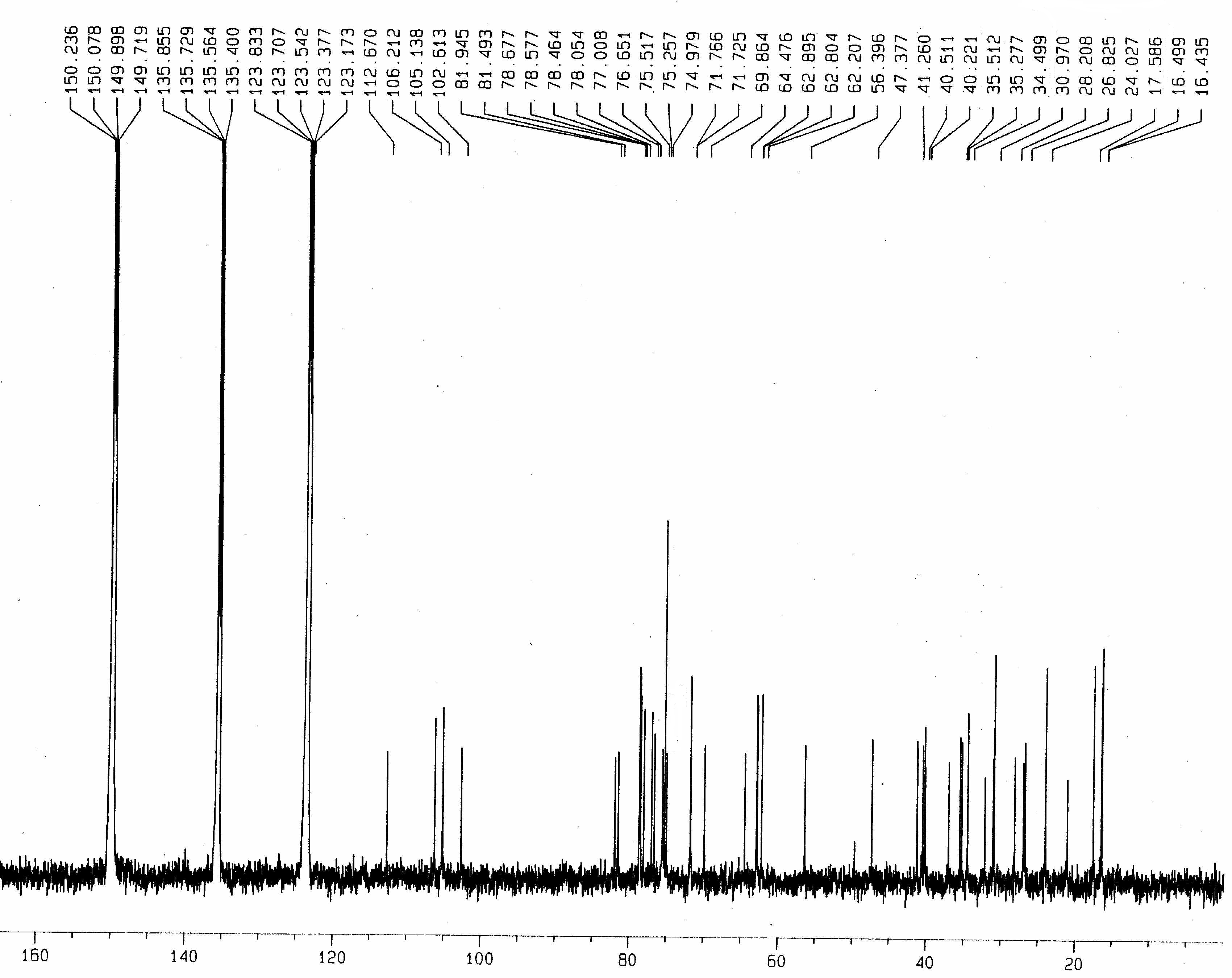


**Compound 20:**


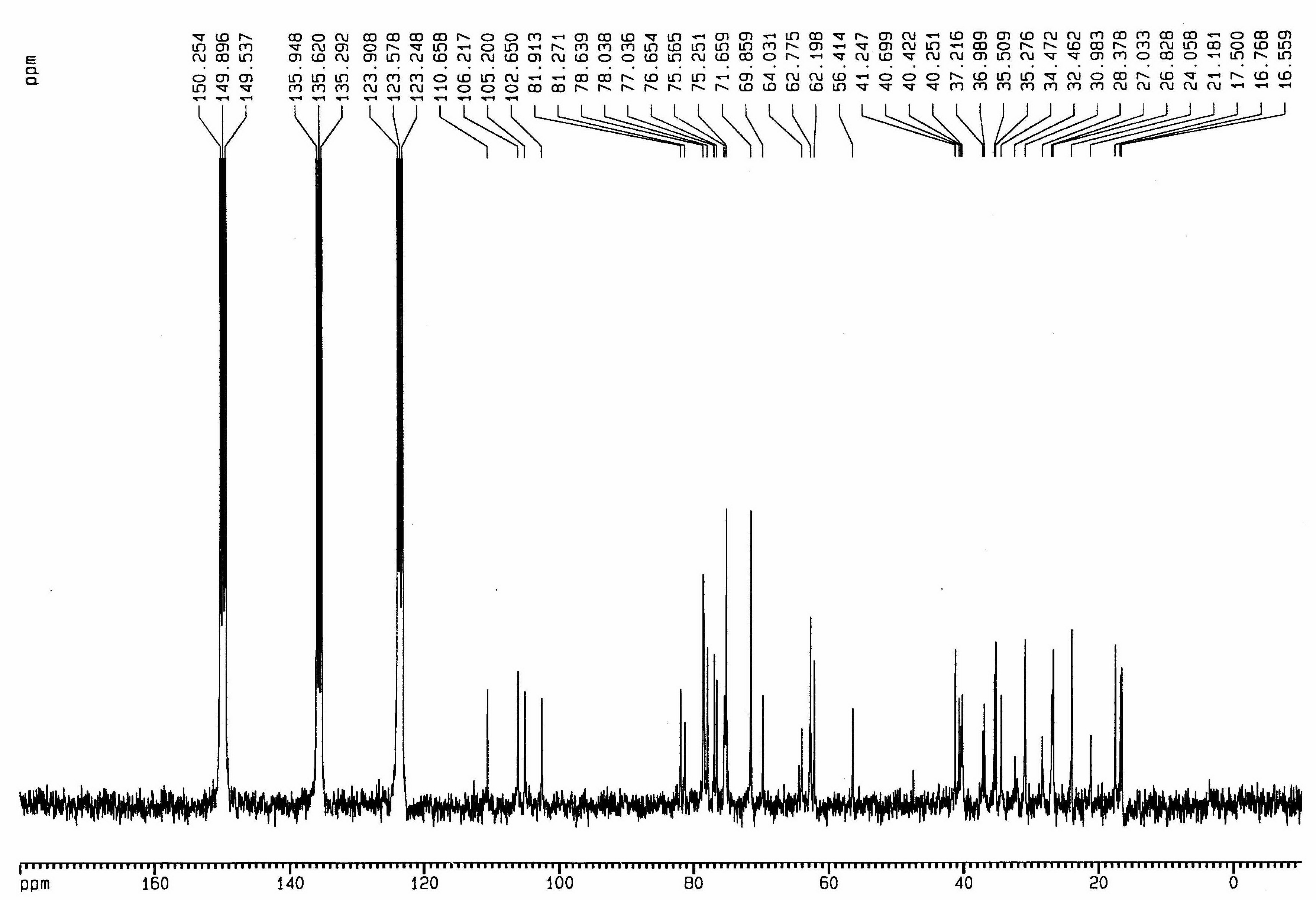


**Compound 21:**


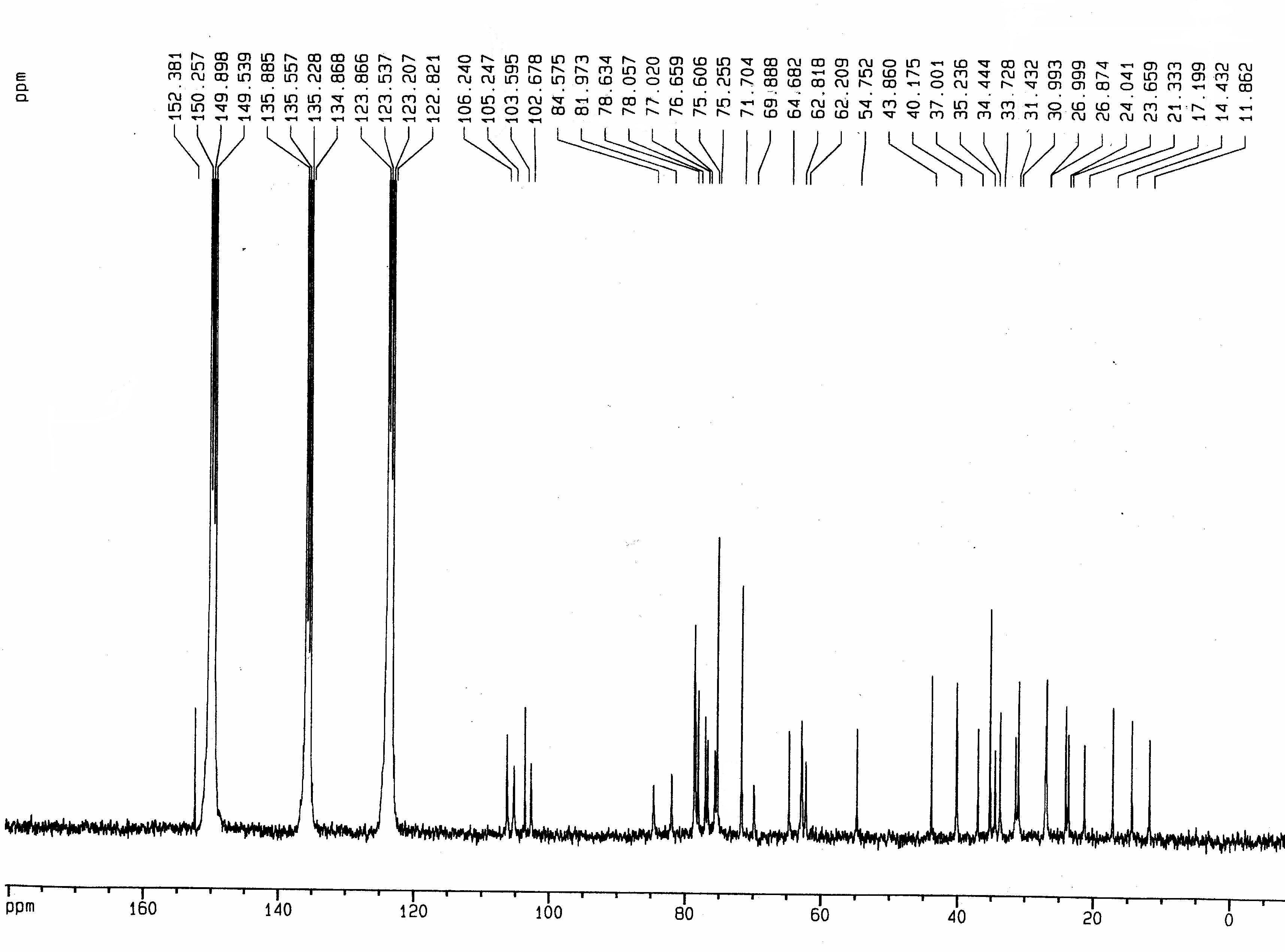


**Compound 22:**


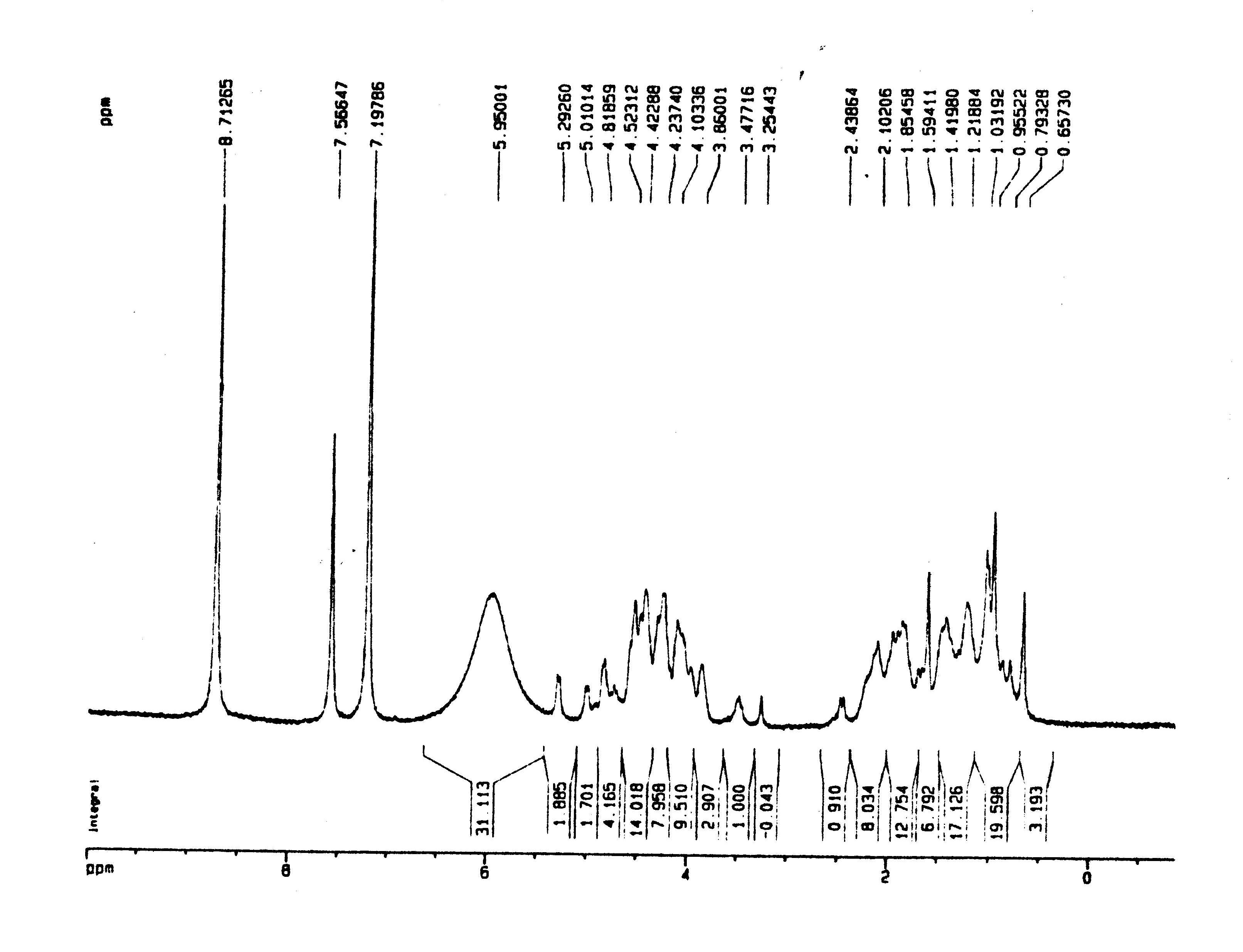

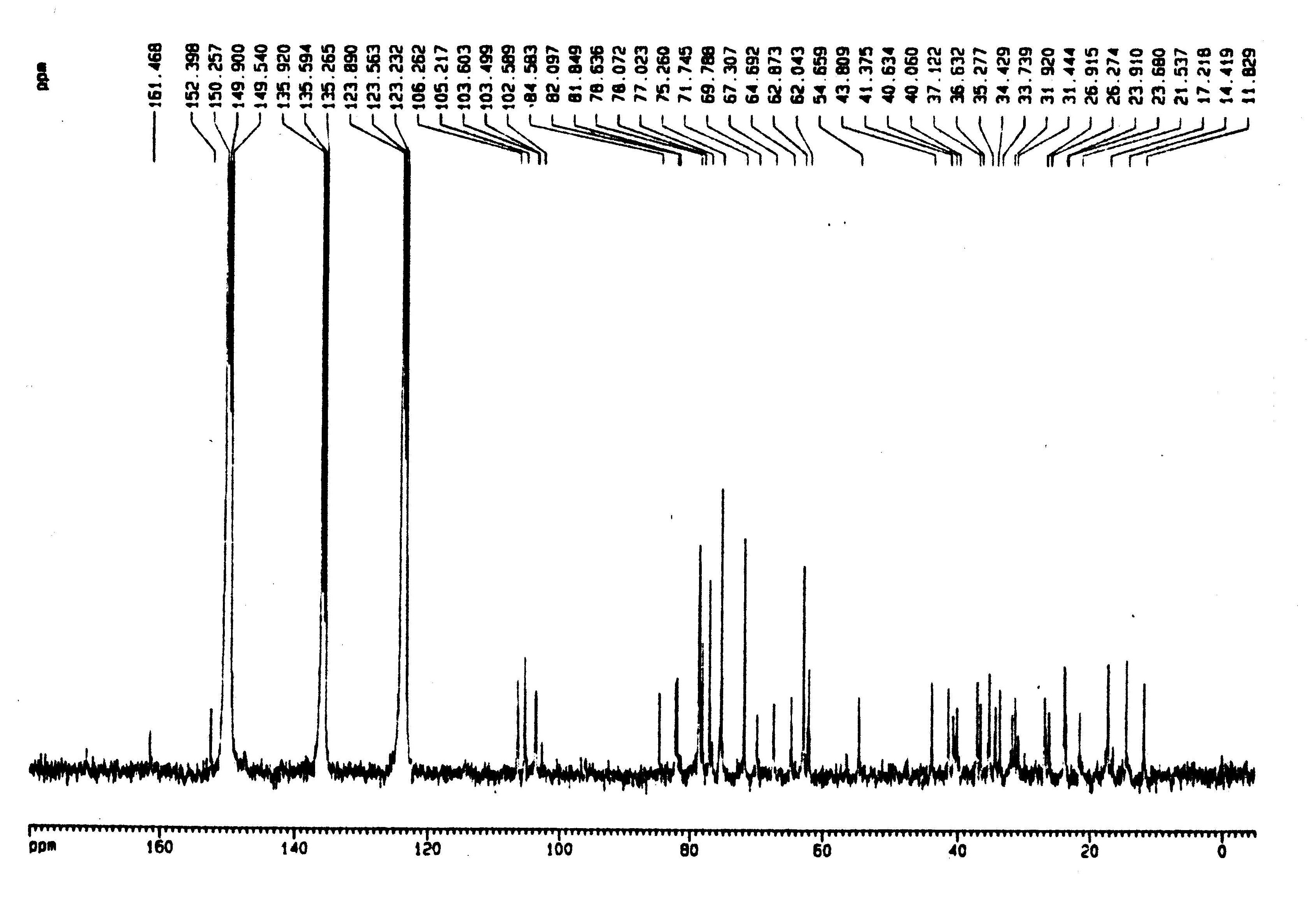


**Compound 23:**


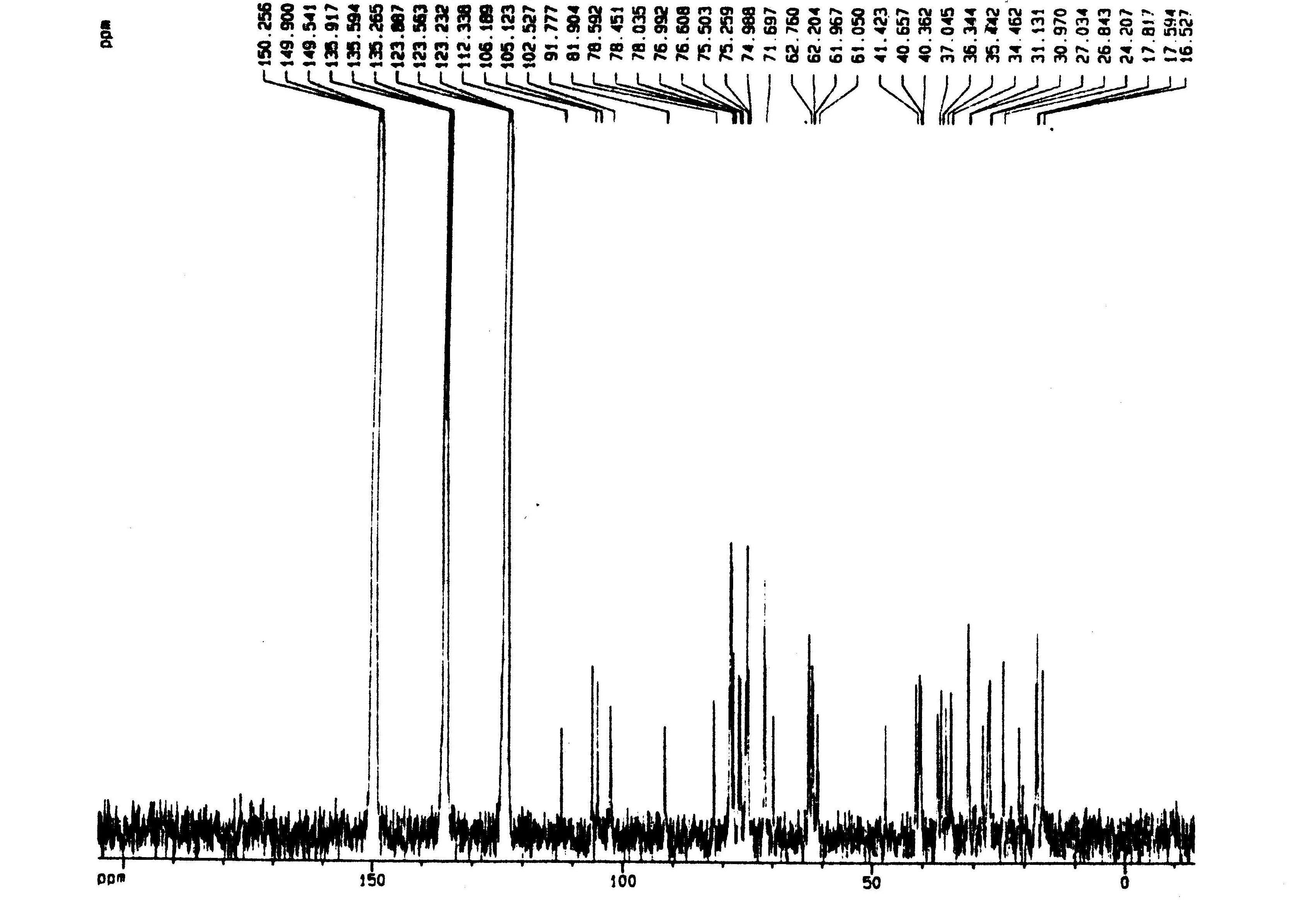


**Compound 24:**


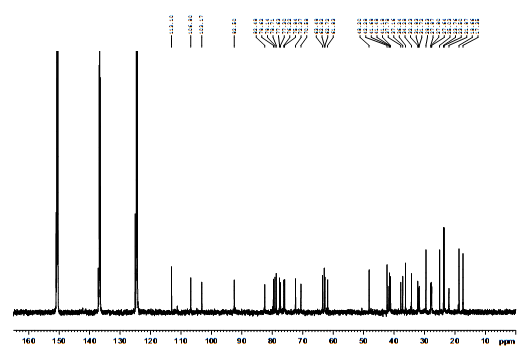


**Compound 25:**


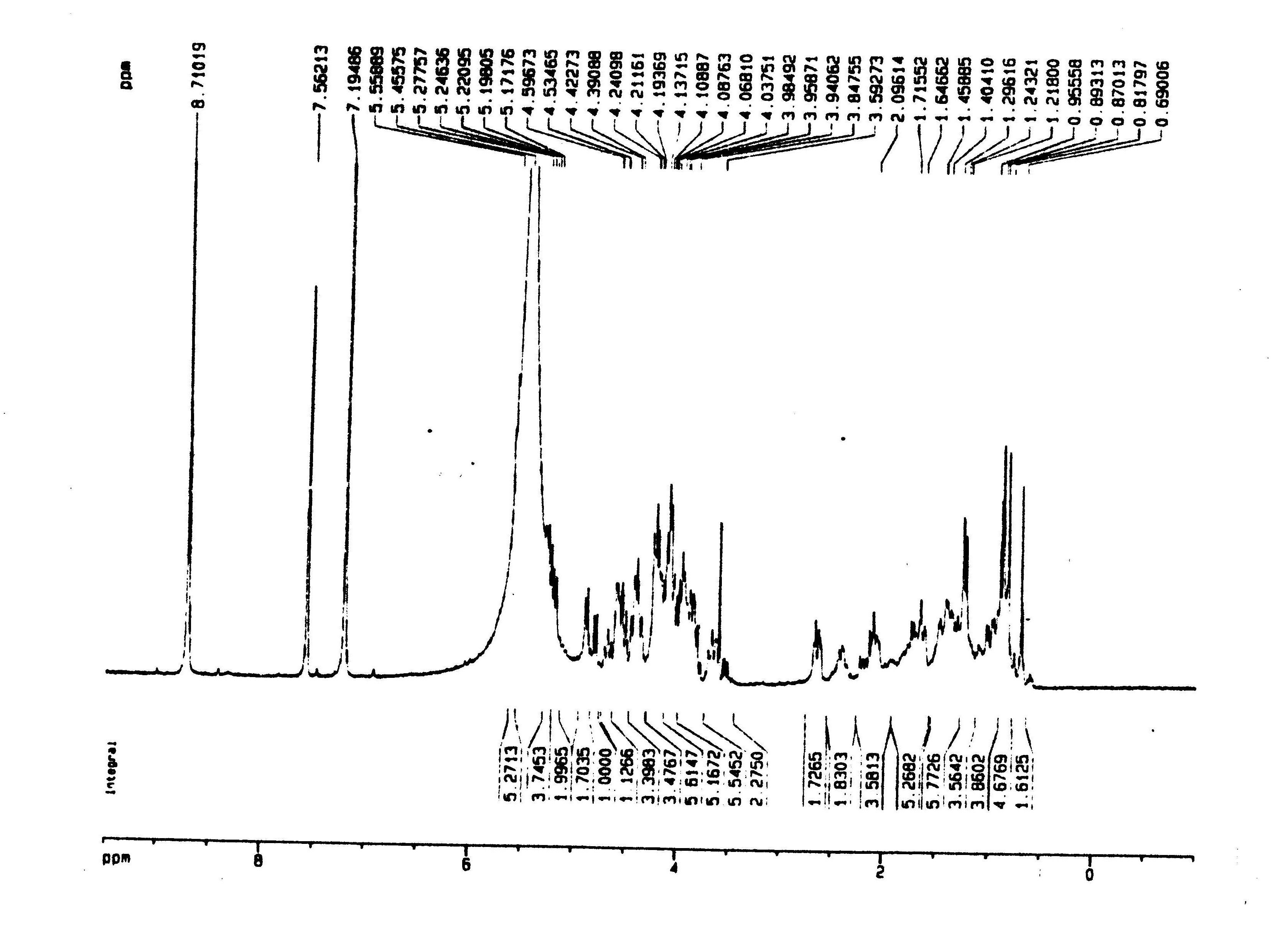

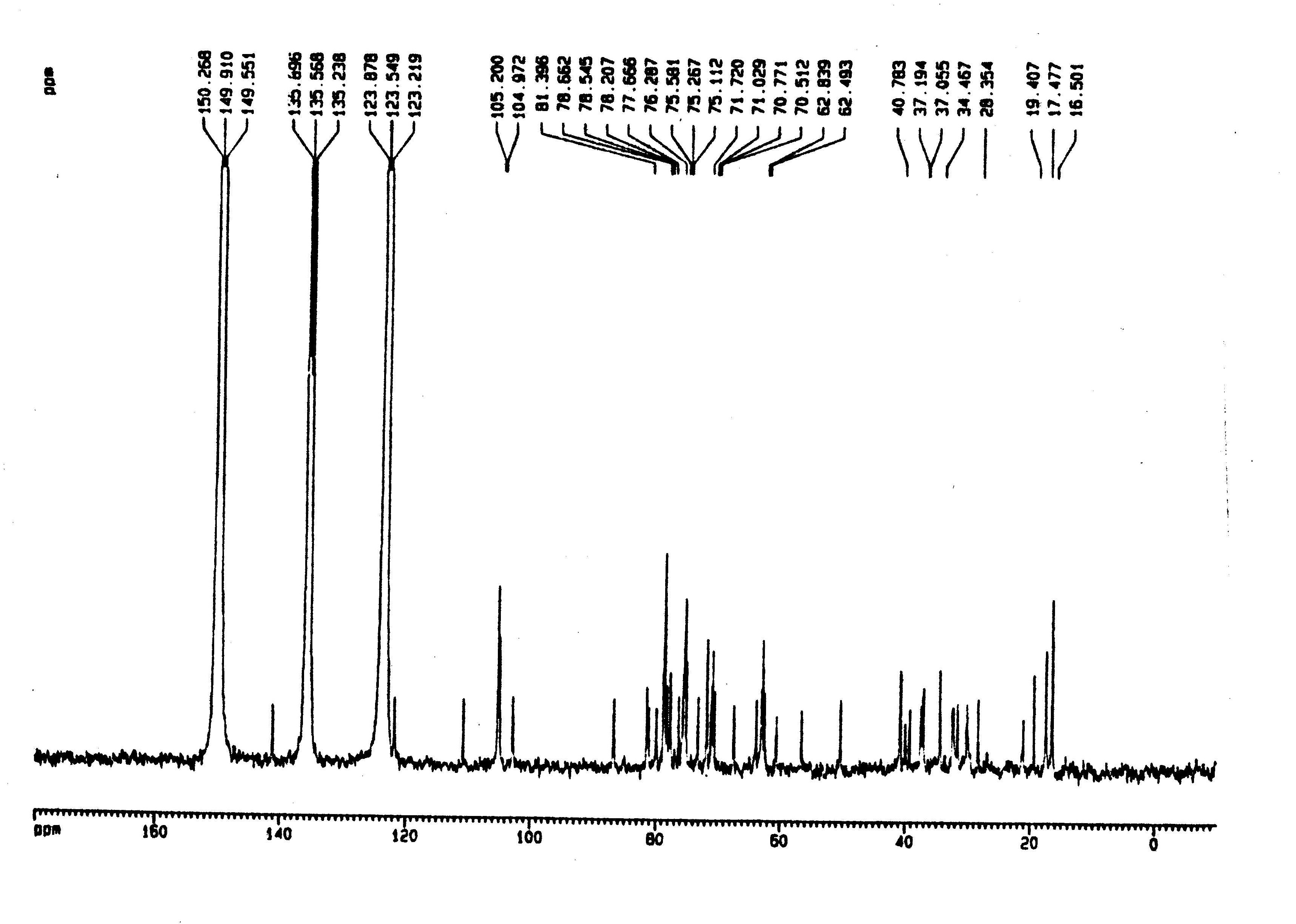

Supplement: Supplementary file 1 — Twenty-five compounds were obtained after repeated isolation and purification from the active fraction. Their structures were established by detailed spectral studies and by the comparison with literature data. The main spectrum of the isolated compounds were exhibited in the supplementary material. [file 524650.f1.doc]
